# Large-scale benchmarking reveals false discoveries and count transformation sensitivity in 16S rRNA gene amplicon data analysis methods used in microbiome studies

**DOI:** 10.1186/s40168-016-0208-8

**Published:** 2016-11-25

**Authors:** Jonathan Thorsen, Asker Brejnrod, Martin Mortensen, Morten A. Rasmussen, Jakob Stokholm, Waleed Abu Al-Soud, Søren Sørensen, Hans Bisgaard, Johannes Waage

**Affiliations:** 1 COPSAC, Copenhagen Prospective Studies on Asthma in Childhood, Herlev and Gentofte Hospital, University of Copenhagen, Copenhagen, Denmark; 2 Section of Microbiology, Department of Biology, University of Copenhagen, Copenhagen, Denmark; 3 Department of Biology, Laboratory of Genomics and Molecular Biomedicine, University of Copenhagen, Copenhagen, Denmark

**Keywords:** 16S sequencing, Microbiome, Benchmark, Differential relative abundance, Beta-diversity

## Abstract

**Background:**

There is an immense scientific interest in the human microbiome and its effects on human physiology, health, and disease. A common approach for examining bacterial communities is high-throughput sequencing of 16S rRNA gene hypervariable regions, aggregating sequence-similar amplicons into operational taxonomic units (OTUs). Strategies for detecting differential relative abundance of OTUs between sample conditions include classical statistical approaches as well as a plethora of newer methods, many borrowing from the related field of RNA-seq analysis. This effort is complicated by unique data characteristics, including sparsity, sequencing depth variation, and nonconformity of read counts to theoretical distributions, which is often exacerbated by exploratory and/or unbalanced study designs. Here, we assess the robustness of available methods for (1) inference in differential relative abundance analysis and (2) beta-diversity-based sample separation, using a rigorous benchmarking framework based on large clinical 16S microbiome datasets from different sources.

**Results:**

Running more than 380,000 full differential relative abundance tests on real datasets with permuted case/control assignments and in silico-spiked OTUs, we identify large differences in method performance on a range of parameters, including false positive rates, sensitivity to sparsity and case/control balances, and spike-in retrieval rate. In large datasets, methods with the highest false positive rates also tend to have the best detection power. For beta-diversity-based sample separation, we show that library size normalization has very little effect and that the distance metric is the most important factor in terms of separation power.

**Conclusions:**

Our results, generalizable to datasets from different sequencing platforms, demonstrate how the choice of method considerably affects analysis outcome. Here, we give recommendations for tools that exhibit low false positive rates, have good retrieval power across effect sizes and case/control proportions, and have low sparsity bias. Result output from some commonly used methods should be interpreted with caution. We provide an easily extensible framework for benchmarking of new methods and future microbiome datasets.

**Electronic supplementary material:**

The online version of this article (doi:10.1186/s40168-016-0208-8) contains supplementary material, which is available to authorized users.

## Background

Technical advances in DNA sequencing have allowed for the collection of high-dimensional biological data on an unprecedented scale. This development has ignited a surge of scientific opportunities and interest in the human microbiome and its effects on human physiology, health, and disease [1, 2]. A common approach to microbiome studies is the amplification of hypervariable regions of bacterial 16S rRNA genes from biological samples, sequencing of amplicons in a high-throughput fashion, and grouping of sequences into operational taxonomic units (OTUs) [3–5] for downstream applications.

A common statistical analysis of OTU data is differential relative abundance (DA) testing, a serial univariate test of each OTU between two sample groups, e.g., phenotypes, compartments, or time-points. This relatively simple endeavor is complicated by certain characteristics of the data, in particular three major points. First, the OTU count matrix is sparse, with often between 80 and 95% of the counts being zero [6, 7]. Second, the library sizes (sum of counts in each sample; also referred to as sequencing depth) vary significantly, sometimes by several orders of magnitude, making it nonsensical to compare counts directly between samples, since they each represent a different fraction of the composition of a given sample. Third, as is well known from ecological literature, the variances of these count distributions are greater than their means, a phenomenon known as overdispersion [8, 9]. In the RNA-seq field which is based on similar sequencing technology, explicit modeling of this mean-variance relationship has been attempted [10, 11].

The aim of this work is to benchmark the many options investigators face when analyzing 16S amplicon-based sequencing data. Previous work with similar objectives has focused on the practice of rarefaction [12, 13], i.e., resampling reads within each sample to equal amounts to overcome the differences in sequencing depths. This work attempts three separate benchmarks of inference robustness, all based on real datasets generated from clinical samples, obtained from different compartments in the human microbiome, including the gut, hypopharynx, and vagina, covering a wide range of human ecological niches.

First, we have quantified the false discovery rate of the most popular differential relative abundance (DA) methods by randomly assigning case/control status to samples, thus creating an empirical null distribution, and testing each OTU for differential relative abundance. Second, we have simulated in silico spiking of known magnitudes and examined how well these can be recovered. We have used a range of multiplicative and additive spike-in magnitudes applied to OTUs from different relative abundance tertiles to explicitly control the range of OTUs to be recovered. Furthermore, as the microbiome field is currently in a state where many projects are exploratory and not explicitly designed, we have examined the effect of the case/control proportion. The included methods are well-established choices for data analysis in many fields. The Welch two-sample *t* test is the default choice for comparing two sample means, while the Wilcoxon rank sum test is a nonparametric alternative. Negative binomial generalized linear models (GLM) have long been a popular option in ecology for modeling count data such as species observation counts [14], by adding an additional parameter to account for the aforementioned overdispersion. From the field of RNA-seq, which have faced many of the same data analysis challenges, we have included two widely used packages estimating counts parametrically, also utilizing the negative binomial distribution, DEseq2 [15] and edgeR [16], as well as baySeq [17], using an empirical Bayes method for parameter estimation. From the field of microbial ecology, metagenomeSeq [7] has been designed with microbial marker surveys in mind, using a normalization procedure and a zero-inflated gaussian (ZIG) mixture model, designed to handle sequence depth issues and sparsity, as well as an alternative zero-inflated log-normal model with included parameter shrinkage (feature model) [18]. The ALDEx2 method has been developed with emphasis on the compositional nature of sequencing data, implementing Monte Carlo sampling of Dirichlet distributions and averaging *p* values across resamples [19]. In addition, we have implemented a simple custom permutation test, based on the null distribution of a test statistic defined as $\log {\left(\frac{\mathrm{mean}\ \mathrm{counts}\ \mathrm{in}\ \mathrm{cases}}{\mathrm{mean}\ \mathrm{counts}\ \mathrm{in}\ \mathrm{controls}}\right)}^2$ obtained through random permutations of samples as cases/controls. Finally, we have quantified the effect of normalization, transformation, and choice of distance measure on the beta-diversity separation of samples with a known biological grouping. Multivariate analysis and choice of distance measure in particular are currently being debated in microbial ecology as claims of inherent clustering of vaginal [20] and gut microbiomes [2] have been made. The robustness of these claims has been shown to be sensitive to data analysis choices [21], visualization choice [22], and copy number estimation procedures [23]. Ecology has a long tradition for multivariate analysis of species tables, and many of the currently available tools have therefore been adapted from this field, such as the Bray-Curtis dissimilarity measure [24]. Microbial ecology has seen the development of measures exploiting phylogenetic information in the sequencing reads. Here, we include the weighted and unweighted UniFrac [25, 26]. Additionally, we included the Jensen-Shannon divergence, which plays a key role in enterotyping and clustering of vaginal microbiomes. The Euclidean distance is known to be unsuited for ecological distance measurements due to what has been termed the “double zero” problem, the fact that it is not possible to distinguish if a species is absent from two samples due to undersampling [7, 27]. It has been included as a baseline, since many early papers explored microbiomes using Euclidean-driven principal component analysis.

## Results

### Study design and data characteristics

**Fig. 1:**
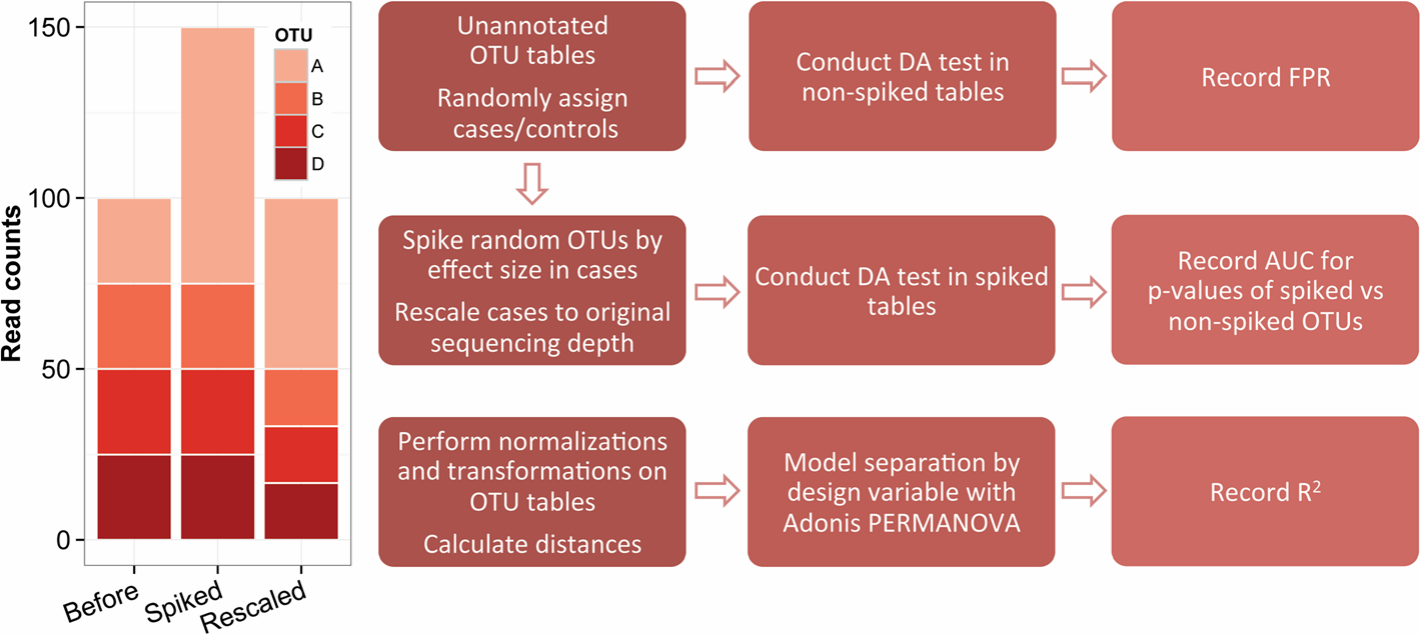
Spike-in approach and analysis flowchart. *Left*, a theoretical sample where the count data for OTU A is multiplied by 3 before rescaling to original sequencing depth. *Right*, flowchart of the simulation study, yielding FPR and AUC for each method, dataset, and set of variables, as well as *R* ^2^ values for all combinations of normalization, transformation, and distance in the beta-diversity optimization

The study was divided into three parts (Fig. 1), namely (1) false positive rate (FPR) testing, (2) spike-in retrieval testing, and (3) beta-diversity optimization. We used seven large datasets: three for the FPR tests and spike-in retrieval tests (labeled A1-A3), one simulated set (labeled A4) for assessing the effect of spike-in independent sample strata, and three for the beta-diversity optimization tests (labeled B1–B3). The datasets and their characteristics are presented in Additional file 1: Table S1. The datasets are characterized by having high degrees of sparsity, large variation in library sizes, and an overdispersed mean-variance relationship (Additional file 2: Figure S1).

### False positive rates

We found striking differences in the FPR of the tested methods using identical permutations of the three large datasets A1–A3 (Additional file 3: Figure S2A). A total of 17,550 full DA tests were analyzed. Generally, many methods were robust, with FPR close to or below 0.05, as expected under the null hypothesis. However, edgeR, metagenomeSeq ZIG (unfiltered, see below), and especially baySeq displayed very high FPRs, indicating that models did not fit well to the data. Intriguingly, baySeq, edgeR, and negative binomial GLMs performed worse under balanced conditions, i.e., 50% cases and 50% controls, than under unbalanced conditions with only 10% cases. Most methods had low variance of FPR across iterations, but metagenomeSeq ZIG and especially baySeq showed considerable variation within parameter sets. To ensure that observed differences in FPRs between balanced conditions were not due to inherent biological signals or sample structures in the datasets used, we repeated the analysis in an additional simulated dataset (A4, *n* = 5850), based on within-OTU count permutation, retaining the biological distribution of OTU count data but breaking within-sample characteristics. With the exception of baySeq, no major deviations were observed from the results obtained with dataset A3 (Additional file 3: Figure S2B).

**Fig. 2:**
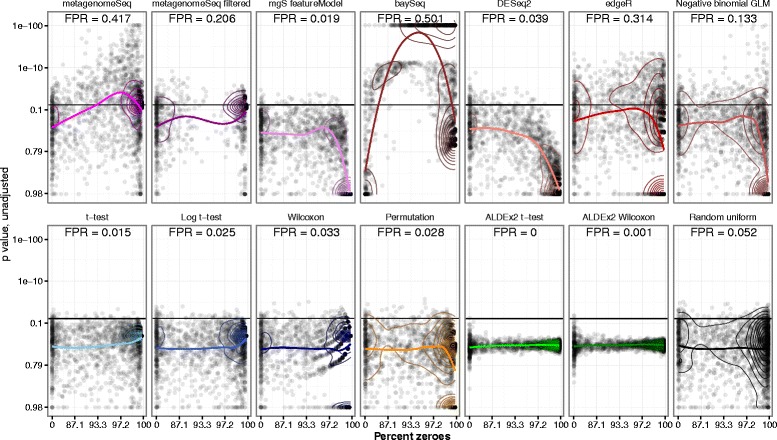
OTU sparsity vs. *p* value. Scatterplots of OTU sparsity vs *p* value with panels representing each differential relative abundance test method in feces dataset A1, with 50% cases. *Colored line* represents the LOESS regression on data. False positive rate (FPR) is defined as the fraction of OTUs with *p* < 0.05. Each differential relative abundance test represents the median FPR for that method, out of all 150 permutations. *Contour lines* indicate point density and can be compared to a hypothetical null distribution of *p* values demonstrated in the final panel (“Random uniform”)

Next, we investigated the effect of OTU sparsity on test inference (Fig. 2) and observed that the sparsity of any given OTU had different effects when applying the different methods, in the feces dataset A1. OTU-wise *p* values from non-spiked single DA runs with 50% cases, selected by the median FPR, depended to some extent on the percentage of zeroes in the OTU in question. The methods edgeR, negative binomial GLM, metagenomeSeq ZIG (unfiltered), and especially baySeq displayed biased results at high sparsity, meaning that many zeroes lead to lower *p* values, irrespective of any signal in the data. This effect was to some extent ameliorated for metagenomeSeq by its filtering step, which essentially removes most of the sparse OTUs after the model has been estimated, as demonstrated in Fig. 2. The feature model did not exhibit this inflation. DESeq2 in particular exhibited a conservative estimation of *p* values at high sparsity and *t* tests, and Wilcoxon and the permutation tests were all very robust across the range. The ALDEx2 method was very conservative and showed a narrow band of *p* values resembling a Gaussian distribution around 0.5, regardless of sparsity.

### Spike-in retrieval tests

A total of 175,500 spiked DA analyses from datasets A1–A3 were considered. The spike-ins were performed by increasing the raw counts of random OTUs in cases, either multiplicatively or additively (see Fig. 1), by a range of spike-in magnitudes, in order to measure the relative retrieval performance between methods.

Additional file 4: Figure S3A shows the area under the curve (AUC) value distributions of the receiver operating characteristics (ROC) curve when using *p* values to discriminate between multiplicatively spiked and non-spiked OTUs in the feces dataset A1, for all the methods, case proportions, and magnitudes. We found that most methods improved detection power as the spike-in magnitudes increase, though both Wilcoxon and metagenomeSeq ZIG (filtered) did not exhibit this property to the same extent as the others. The multiplicative spike-in AUC distributions for the other two datasets A2 and A3 (Additional file 4: Figure S3B, S3C) showed very similar characteristics. Overall, the best performance in terms of AUC was exhibited by the sequencing-specific methods edgeR, metagenomeSeq ZIG, and baySeq, as well as the assumption-free permutation test. The mid-level in performance was represented by negative binomial GLM, DESeq2, and ALDEx2, whereas *t* tests and Wilcoxon performed the worst. The robustness of these methods varied greatly, with some tests yielding AUC values below 0.5, in case of the *t* tests and Wilcoxon even with a median value below 0.5 in the unbalanced tests with case proportions at 10%. The most robust test was the permutation test and metagenomeSeq feature model, which only very rarely fell below 0.5 in AUC.

We repeated these analyses with additive spiking (Additional file 5: Figure S4A, S4B, S4C), yielding very similar results, albeit with lower variance in the distributions. We found that the methods exhibited the same hierarchy of performance across the three datasets as in the multiplicative spike-in tests. The best performing models were saturated already at magnitude 10, rendering magnitude of change 20 unnecessary.

Finally, we considered a mixed spike-in setup (Additional file 6: Figure S5). In this setup, methods were not as clearly separated in AUC values, although the same general hierarchy was retrieved. The highest AUC values were found in the methods edgeR, metagenomeSeq ZIG, negative binomial GLMs, and the permutation test.

**Fig. 3:**
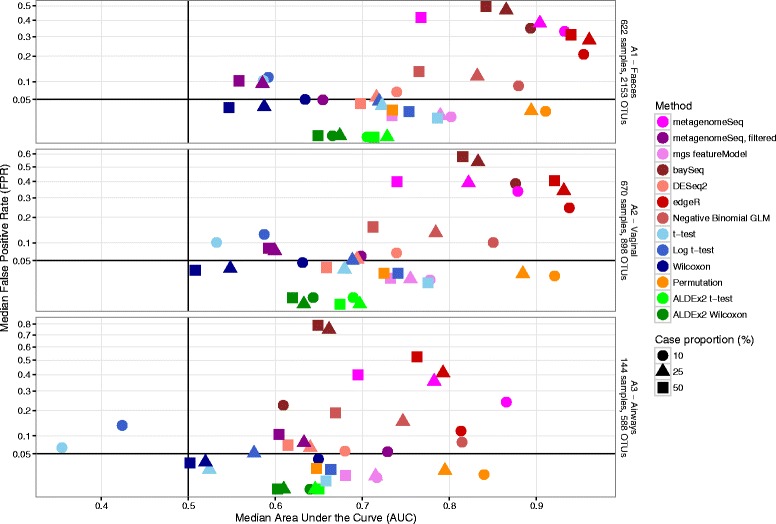
Median test AUC vs median test FPR. Scatterplots of median test area under the curve (AUC) vs median test false positive rate (FPR), for the datasets A1–A3; three different compartments in the COPSAC_2010_ cohort. FPR is defined as the fraction of OTUs with *p* < 0.05. *Dot color* represents differential relative abundance test method, while *dot shape* represents experiment case/control balance

Figure 3 shows the median AUC values vs the median FPRs, illustrating the overall performance of the various methods in the three datasets, at the highest magnitude (20) with multiplicative spiking.

### Subsets and simulated data

**Fig. 4:**
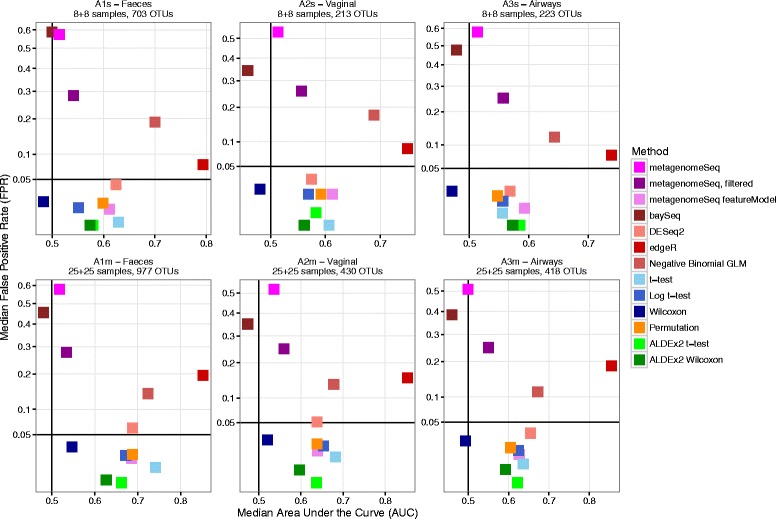
Median test AUC vs median test FPR, small and medium subsets. Scatterplots of median test area under the curve (AUC) vs median test false positive rate (FPR), for the datasets A1s–A3s and A1m–A3m; three different compartments in the COPSAC2010 cohort, with 50% case/control proportion. FPR is defined as the fraction of OTUs with *p* < 0.05. *Dot color* represents differential relative abundance test method

We repeated the FPR (*n* = 11,700) and spike-in tests (*n* = 117,000) to examine the relative performance of the various methods in small- and medium-sized datasets by subsetting datasets A1–A3, yielding datasets of lower sparsity (A1s–A3s and A1m–A3m) (Additional file 1: Table S1). We found that the performance hierarchy was very similar across all six subsets with regard to both FPR and spike-in AUC (Fig. 4). There were some deviations from the larger sets, especially metagenomeSeq ZIG and the permutation test performed worse in the subsets, whereas edgeR kept a high AUC but with much lower FPR. The distributions of FPR and AUC under all parameter combinations can be seen in Additional file 7: Figure S6A–D.

In the simulated dataset A4, the AUC results (*n* = 58,500, Additional file 8: Figure S7A–C) were nearly identical to those from dataset A3, on which it was based, except for baySeq, which performed worse in dataset A4, but with very high variability.

### Spike-in retrieval sensitivity to sparsity

We examined the effect of OTU sparsity on the ability of the various methods to retrieve spiked OTUs, expressed as the *p* value quantile of a spiked OTU within all *p* values from a dataset, for each method, which should ideally be as low as possible. Additional file 9: Figure S8A–C shows that almost all methods had better detection power at low sparsity (many positive samples), but the patterns of saturation were quite different. Additional file 9: Figure S8A shows that most methods were primarily dependent on the number of positive samples, with the lines from the differently sized datasets following each other closely. Notably, Wilcoxon tests were negatively influenced by zero-inflation, meaning that the detection power was decreased with dataset size for the same number of positive samples. For the smaller datasets, metagenomeSeq ZIG and baySeq did not show better performance with more positive samples. The filtered metagenomeSeq ZIG and feature model had several OTUs with quantiles of 1, since *p* values were filtered or not computed and therefore set to a value of 1. Additional file 9: Figure S8B shows sensitivity to case proportion, where almost all methods had better performance across the range with more balanced group sizes, though the effect was greatest for metagenomeSeq feature model and the *t* test. Finally, in Additional file 9: Figure S8C, we see that most methods saturate faster with high spike-in magnitude. Notably, the Wilcoxon test gains little from increased magnitudes. Overall, the metagenomeSeq feature model seemed to saturate at the lowest number of positive samples for across these figures, although it required at least two positive samples in each group to compute a *p* value, most evident in Additional file 9: Figure S8B.

### Beta-diversity optimization

**Fig. 5:**
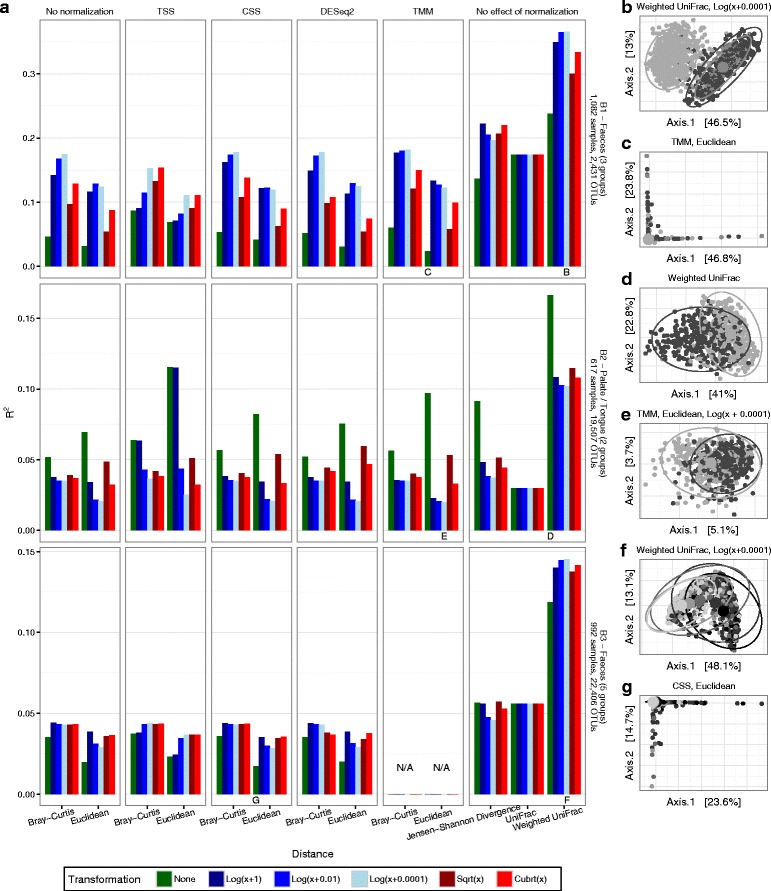
The effect of normalization, transformation, and distance metric on beta-diversity separation. **a** Datasets B1–B3 (*vertical panels*) with all combinations of library size normalizations (*horizontal panels*) and count transformations (*color*) applied prior to the calculation of distances and use of Adonis permanova model. Effect of design variable in question (B1—age; B2—tongue versus palate; B3—age group) measured as model *R* ^2^ value. The highest and lowest *R* ^2^ values (yielding best and worst separation, respectively) are demonstrated in subplots **b**–**g** for each dataset as principal coordinate analysis plots, colored by design variable, with overlaid prediction ellipses (B1—subplot (**b**, **c**); B2—subplot (**d**, **e**); B3—subplot (**f**, **g**)). *CSS* cumulative sum scaling, *TMM* trimmed mean of *M* values, *TSS* total sum scaling

We studied the effects of library size normalization, count transformation, and distance metric on the ability to separate biologically relevant groups in beta-diversity analyses (Fig. 5). All analyses were significant with *p* < 0.001, but with large differences in *R* ^2^ values. In the feces dataset B1, the optimal separation was found in log transformed counts using 10^−5^ as pseudocount with weighted UniFrac, yielding an Adonis *R* ^2^ value of 0.367. In the HMP dataset B2, the optimal was non-transformed weighted UniFrac, with an *R* ^2^ value of 0.166. In the feces dataset B3, TMM normalization was not possible due to high sparsity, and this method was omitted from the analysis. The optimal separation was found in log transformed counts using 10^−5^ as pseudocount with weighted UniFrac, yielding an *R* ^2^ value of 0.145. As a sensitivity analysis to include TMM, we agglomerated closely related OTUs, thereby reducing the number of OTUs and the sparsity, which did not materially change the results, see Additional file 10: Figure S9.

Across all three datasets, several characteristics were very similar. The most important factor was the choice of distance metric, with the weighted UniFrac metric scoring the highest in terms of separation power in all three datasets. The transformation applied to (normalized) counts was of less importance. In the feces dataset B1, log transformations with very small pseudocounts were best, whereas the untransformed counts were optimal in the HMP oral dataset B2. In both cases, the effects were large. The effect of library size normalization was by far the lowest, especially between no normalization, CSS, DESeq2, and TMM normalization, which essentially did not matter in terms of separation in any of the three datasets. TSS was the normalization type with the highest impact, but it mostly changed the optimal transformation choice, rather than improve or deteriorate the separation power as such. These effects were very apparent when comparing the optimal combinations of normalization, transformation, and distance with the poorest for each dataset, as described above, visualized with principal coordinates analysis (PCoA) plots in panels B and C of each dataset subplot.

## Discussion

We conducted extensive benchmarking of the most popular available methods for differential relative abundance testing of large microbiome datasets. The main characteristics of each method in terms of FPR, AUC, balance sensitivity, and computational burden are summarized in Additional file 11: Table S3. We found that several methods, including edgeR, metagenomeSeq ZIG, and baySeq, had high false positive rates when testing randomly permuted data, often grossly overestimating the OTU-wise differences between two groups, which indicates that assumptions made by the models were not met by the data. Intriguingly, the methods with the highest FPR also had the highest AUC values for recovering our spiked OTUs. Thus, the *p* values obtained from these methods are very well suited to distinguish differentially abundant OTUs from non-differentially abundant OTUs, but are not meaningful in relation to normal thresholds for significance (i.e., 5%), instead representing an arbitrary classifier value. This problem could potentially be solved by setting a more restrictive significance threshold, although the value of this threshold would need to be set empirically for each dataset, for instance through a permutation similar to the setup of this study. Even then, for some of the methods, especially metagenomeSeq ZIG and baySeq, we found that *p* values varied greatly with the sparsity of a given OTU, meaning that this empirical cutoff value should not be the same in all OTUs but, to some extent, depend on the sparsity of that OTU to accurately reflect the null distribution. It has to be noted that baySeq was run with the default negative binomial prior distribution, but allows the user to define a custom parameterization for the prior, which could improve the performance of baySeq in this regard. We have not explicitly addressed pre-inference filtering, which is a common practice to reduce the strain of correction for multiple inference. However, we have examined the effects of metagenomeSeq ZIG’s recommended filtering step. We found that this filtering removed the most sparse/rare OTUs, which ameliorates the abovementioned dependence of *p* values on sparsity. However, it is a very conservative filtering, which could also be applied to any of the other methods, and does not fix the underlying problems with the fit of the statistical model. Indeed, many rare OTUs could be truly differentially abundant in many types of studies. We have analyzed crude *p* values across all methods, and not explicitly corrected *p* values for multiple inference, leaving the expected null FPR at 0.05. This correction is a necessary step in most situations and is often done by controlling the false discovery rate using the Benjamini-Hochberg approach [28] or the familywise error rate using, e.g., the Bonferroni correction [29]. However, this step is independent of model choice and should be applied regardless of which method is used to obtain *p* values, which makes it irrelevant in our study setup.

The inclusion of a permutation test is not meant as a recommendation or novel method, but it proves an interesting comparison as it is simple, extremely robust, and has good detection power, such that it far outperforms the other simple methods—*t* test and Wilcoxon. It should be noted that the many ties in sparse data may disproportionately limit the maximum statistical power of rank-based tests such as the Wilcoxon test (illustrated in Additional file 12: Figure S10), which was also evident in Additional file 9: Figure S8A–C.

Furthermore, under some circumstances, the *t* test produced AUC values that were below 0.5, i.e., worse than random performance. As can be seen from the contour lines in Fig. 2, this occurred due to the *t* test producing too low *p* values at extreme levels of sparsity, where only one sample was positive, which overpowered the *p* value decrease from the weaker spike-in magnitudes as these were selected to represent low, medium, and high levels of sparsity. Naturally, this phenomenon can be attributed to unmet distributional assumptions in the data.

The AUC statistic is usually employed as a measure of separation, e.g., how well does a certain biomarker distinguish between healthy and sick. However, it can also be used as a scale-independent enrichment statistic, as in the present study. Importantly, at low spike-in magnitudes, AUC values should not be expected to be close to 1 but should rather be used to compare power between different methods.

We repeated the analyses in small- and medium-sized datasets, since many researchers opt for smaller, balanced designs when testing specific experimental hypotheses. These results showed that some methods performed worse (permutation test, metagenomeSeq ZIG), while others improved (edgeR) when compared to the results from the large datasets. This phenomenon may be linked to the decreased sparsity of these smaller sets, as described in Additional file 1: Table S1, due to a lower amount of rare taxa compared to common taxa, in addition to the differences in statistical power of the methods given low sample sizes, which may limit real-world applications.

Previously, the optimal library size normalization for beta-diversity measures have been thoroughly discussed, and count transformations have been recognized as important approaches for optimally separating biologically meaningful groups [7, 13, 30–32]. Our results highlight the importance of carefully considering which normalizations, count transformations, and distance metrics should be applied to identify the best separation in the beta-diversity space. In particular, the relative impact of these three factors has been elucidated. Generally, we found that library size normalization is the least important of the three. Especially the difference between no normalization, CSS, DESeq2, and TMM normalizations was negligible in all three datasets. TSS normalization was the most different from the others, but mostly changed the optimal transformation choice rather than improve or deteriorate the separation, which highlights the importance of choosing an appropriate pseudocount relative to the scale of the data when using log transformations. Count transformations did not necessarily improve separation but was very impactful in all cases. The effect of a log transform is down-weighting of high-abundant taxa and up-weighting of low-abundant taxa, which is a pivotal consideration in terms of expected abundance levels of taxonomic differences between groups in a study. The most important factor was the choice of distance metric. In all of our examples, the best separation was found with the weighted UniFrac metric. However, this study was not designed to infer which distance metric is best, as this will depend on the data, but rather the relative importance of these three factors. It should also be noted that *R* ^2^ values for presence/absence distance metrics such as unweighted UniFrac may be inherently limited by sparsity and rare taxa. Additionally, the problems with increasing sparsity in large datasets observed in the taxon-wise DA tests should not affect beta-diversity tests, since pairwise sample-wise distances do not depend on taxa absent from both samples.

It is a strength of the study that we, through a large amount of computations, have generated results from combinations of parameters of relevance to the field, namely statistical method, normalization method, case/control ratio, sample size, and spike-in magnitude. It is also a strength of this study that our analyses are conducted on large and biologically diverse human microbiome data. Many large-scale microbiome studies are being conducted and planned presently, with diverse human ecological niches represented. Thus, it is important to survey different body sites, since the uniquely different microbial compositions present may influence the distributional characteristics of the resulting datasets. While the results presented here derive from biological data, our results from the spike-in analyses rely on in silico spike-ins, rather than actual biological signals or wet-lab spike-ins. This approach is both a strength and a limitation, in that it allows very precise manipulation and complete control throughout the experiment, as well as the opportunity for nearly limitless repetition to examine well-resolved distributions of the parameters of interest. Conversely, it does not represent an actual biological signal, and manipulating the data may skew certain distributional qualities present, such as the inherent count ratios between OTUs. Though not feasible in this study, future studies could conduct wet-lab spike-ins to track and compare detection power between packages. However, great care must be taken to control the relative concentrations of original content vs. spike-in content, especially in the case of rare OTUs. Hence, this approach also poses many potential issues that may lead to skewed data not accurately reflecting true biological changes.

In previous studies, the best way to account for variation in library sizes has been discussed with a primary focus on the procedure of rarefying counts that is random within-sample resampling of counts without replacement to an even sequencing depth across all samples [12]. This prevalent approach has been criticized due to discarding of valid data, but others argue that it can be the optimal method in some situations, as uneven library sizes disproportionately affects unweighted distance measures and presence/absence analyses [13]. Since the topic of rarefaction already has been debated in detail, and is currently regarded unfavorably, we chose not to include it in this study.

## Conclusion

﻿This study represents an independent attempt to benchmark various methods for differential relative abundance analysis of count-based microbiome datasets, using real biological large-scale datasets. The results presented here warrant an increased awareness of the potential for spurious findings in differential relative abundance analyses. 16S data poses problems to both parametric and nonparametric statistical models, and new methods should explicitly account for sparsity, which is increased in large datasets. Considering the results presented here as a whole, we recommend researchers choose tools for detecting DA that exhibit low false positive rates, that have good retrieval power across effect sizes and case/control proportions, and that are not biased for these parameters at differing levels of (high) sparsity: metagenomeSeq feature model and the basic permutation test both fulfill these criteria for large and small datasets, and edgeR for small datasets. When exploring beta diversity of microbiome data, analysts should carefully consider their choice of count transformation and distance metric, the latter having the largest impact on results. We have provided all source code and source data necessary to reproduce the results presented in this study, including random seeds for random processes. This will allow other investigators to verify and expand upon our results and aid in selecting the optimal analysis methods given the unique characteristics of their own data. The comparisons can easily be extended to analysis methods not covered in this paper, ensuring that computation time, rather than coding time, should be the main limiting factor.

## Methods

### Sample collection and preparation

For dataset A1, A2, A3, and B1, the primary sample materials were collected from the COpenhagen Prospective Studies on Asthma in Childhood 2010 (COPSAC_2010_) mother-child cohort, following 700 children and their families from pregnancy into childhood, as previously described in detail [33]. In this study, we used fecal samples collected at ages 1 week (*n* = 95), 1 month (*n* = 361), and 1 year (*n* = 622); vaginal swabs collected at week 36 of pregnancy (*n* = 670); and hypopharyngeal aspirates (*n* = 144) collected at acute wheezy episodes in children with persistent wheeze aged 1–3 years, using a soft suction catheter passed through the nose. DNA was extracted using MoBio PowerSoil kits on an EpMotion 5075, amplified using a two-step PCR reaction with forward and reverse 16S V4 primers, and sequenced using 250bp paired-end sequencing on an Illumina MiSeq. A full description of the laboratory workflow and the bioinformatics pipeline is available in the Additional file 13.

To examine effects in smaller datasets, we subset datasets A1, A2, and A3 into 16 (small) and 50 samples (medium) by random sampling with recorded random seeds, resulting in datasets A1s–A3s and A1m–A3m. Additionally, we created a simulated dataset A4 by independent resampling of all OTUs across samples, without replacement, of dataset A3.

Additionally, for dataset B2, we used public data from the Human Microbiome Project [34], testing separation ability between the tongue dorsum (*n* = 316) and hard palate (*n* = 301) 16S V3-5 samples (http://hmpdacc.org/HMQCP/). For dataset B3, we used data from Pop et al. [35], downloaded from Bioconductor (http://bioconductor.org/packages/release/data/experiment/html/msd16s.html), testing separation between age groups 0–6 months (*n* = 112), 6–12 months (*n* = 308), 12–18 months (*n* = 173), 18–24 months (*n* = 146), and 24–60 months (*n* = 253).

To reduce sparsity of dataset B3, chimeras were rechecked using USEARCH v7.0.1090 [36] against the gold database [37], and 3624 chimeras (listed in Additional file 14: Table S2) were removed from the OTU table. Since a phylogenetic tree file was not published along with the OTU table and sample metadata from this paper, we built one using the supplied reference sequences as described in the “Bioinformatics” section of the Additional file 13. Due to issues with TMM normalization of this dataset (see the “Results” section), we agglomerated similar OTUs to reduce the sparsity as a sensitivity analysis. This was achieved by computing pairwise phylogenetic distances using the tree and grouping together all OTUs who were closer to each other than the 0.001 quantile of the distance distribution, see Additional file 1: Table S1. The OTUs were merged with the merge_taxa function in the R package phyloseq [38], using the OTU with the highest sum of counts as archetype.

### Differential relative abundance testing

We selected several widely used methods for differential relative abundance testing to apply on the datasets, using built-in library size normalization, default parameters, and testing as applicable for each method. All tests were conducted using the statistical software package R [39] and parallelized using custom bash scripts with GNU parallel [40]. The source code for all testing procedures is available in an online repository. The selected methods and associated transformation steps were as follows:

- metagenomeSeq ZIG [7]: using raw counts, cumulative sum scaling (CSS) was applied with the quantile supplied by cumNormStat. Testing was done using fitZig.
- metagenomeSeq ZIG, filtered: as above, but discarding all OTUs below median effective sample size (supplied by the fitZig model), as recommended by the authors in the package vignette.
- mgS featureModel: as with metagenomeSeq, but using fitFeatureModel to test, rather than fitZig.
- baySeq [17]: using raw counts, two models were defined; no changes and cases vs controls. Library sizes were supplied to the object. Priors were estimated with the negative binomial distribution before estimating likelihoods for cases vs controls. *P* values were defined as 1-likelihood.
- DESeq2 [15]: using raw counts, geometric means were calculated manually and supplied to the estimateSizeFactors function. Standard testing was invoked with DESeq.
- edgeR [16]: using raw counts, normalization factors were calculated with trimmed mean of *M* values (TMM), common and tagwise dispersions were estimated, and testing was done with exactTest.
- Negative binomial generalized linear model (GLM): using raw counts, a model was fitted to each OTU with glm.nb from the MASS package [41], using log (library size) as offset and cases vs controls as the only dependent variable.
- *t* test: counts were transformed to relative abundances, before applying the R built-in t.test function using default parameters, including the Welch/Satterthwaite approximation to degrees of freedom due to possibly unequal variances.
- Log *t* test: as above, but counts were log transformed first, with a pseudocount of 1.
- Wilcoxon: counts were transformed to relative abundances, before applying the R built-in Mann-Whitney/Wilcoxon rank sum test with wilcox.test using default parameters.
- Permutation: a simple custom permutation test was written and applied to counts normalized to relative abundances. First, a test statistic was computed as $\log {\left(\frac{\mathrm{mean}\ \mathrm{counts}\ \mathrm{in}\ \mathrm{cases}}{\mathrm{mean}\ \mathrm{counts}\ \mathrm{in}\ \mathrm{controls}}\right)}^2$. Next, 10^4^ permutations of this test statistic was calculated by random resampling of all cases and controls without replacement to empirically estimate the null distribution of each OTU. The *p* value associated with an OTU was calculated as the proportion of permuted test statistics equal to or greater than the real test statistic.
- ALDEx2 [19]: raw counts were supplied to the aldex function, using 32 Monte Carlo samples, and both the Welch *t* test (we.ep) and Wilcoxon (wi.ep) *p* values were used.

If a method did not return a *p* value for any given OTU, it was set to 1.

### False positive rates

Unannotated OTU tables were tested for FPR by randomly selecting samples as cases or controls, thus assuring the null hypothesis, with varying case proportions of 10, 25, or 50%, and subsequently applying all the abovementioned DA methods. This was repeated 150 times using unique recorded random seeds, identical between methods. A false positive was defined as an OTU with a crude *p* value below 0.05. The FPR was defined as number of false positive OTUs/total number of OTUs.

### Spike-in retrieval performance

Unannotated OTU tables were randomized as described above. Then, five random OTUs from each relative abundance tertile were modified (“spiked”) with a given magnitude, only in cases, to induce a signal in the data. Only OTUs present in at least one of the assigned case samples were eligible. Spiking was done either by multiplying counts by a given magnitude (multiplicative), multiplying by a range of magnitudes (mixed multiplicative), or adding the mean proportion of nonzero counts multiplied by a magnitude to all non-zero counts (additive). After spiking, samples were rescaled to original sequencing depth. This was repeated for the magnitudes 0.5, 2, 5, 10, and 20 for multiplicative and 0.5, 2, 5, and 10 for additive, with the case/control proportions 10, 25, and 50%. All DA methods were applied, and *p* values were obtained as described above. This was repeated 150 times for all combinations of case proportion, spike method, magnitude, and method on each dataset. The area under the receiver operating characteristic curve (AUC) value was calculated using raw *p* values, assuming they were lower in spiked vs. non-spiked OTUs, with the “pROC” package [42].

### Beta-diversity optimization

To assess the effects of normalization, transformation, and distance metrics on the ability of beta-diversity analysis to distinguish between groups, we selected datasets with less-than-perfect separation by a particular design variable. Next, datasets were subjected to different normalization methods (none (included as baseline), total sum scaling/relative abundance (TSS), metagenomeSeq’s cumulative sum scaling (CSS), edgeR’s trimmed mean of *M* values (TMM) and DESeq2’s size factors), and transformations (natural logarithm with pseudocount 1, 0.01, and 0.0001, square root, and cubic root). In case of pseudocounts below one, all post-transformation values were corrected by subtracting the log of the pseudocount, effectively preserving zeroes from the original counts. Finally, selected distance metrics were applied (Bray-Curtis, Euclidean, Jensen-Shannon Divergence, UniFrac, weighted UniFrac) to provide beta-diversity distance matrices from all these combinations. Jensen-Shannon Divergence, UniFrac, and weighted UniFrac are independent of normalization by design and were only computed once per dataset and transformation. We then fitted a distance-based permutation multiple analysis of variance (PERMANOVA) model (Adonis from the R package vegan [43]) to assess the separation power of the given design variable in the dataset, measured as an *R*-squared value. In datasets B1 and B2, where the design included repeated measurements, these were supplied to the model in the strata argument. All data handling and distance calculations were done using the statistical software package R [39] with the add-on package phyloseq [38]. All plots were produced with the package ggplot2 [44].

## Additional files

Additional file 1: Table S1. Overview of the datasets used in the study. Sampling and data characteristics of the seven datasets used in the study, A1–A4 for the false positive rate and spike-in retrieval tests and B1–B3 for the beta-diversity optimization tests. (XLSX 5 kb)
Additional file 2: Figure S1. Data characteristics for feces dataset A1. (A) Dot plot overview of the dataset; black if an OTU was present, blank if not present in a given sample. Sparsity in the dataset is 90.8%. (B) Library size distribution showing differences of several orders of magnitude. (C) Mean-variance relationship showing overdispersion, i.e., higher variance than mean value of a given OTU. (PDF 504 kb)
Additional file 3: Figure S2. A. False positive rate distributions for datasets A1–A3. Violin plot of distributions of false positive rate (FPR) in 150 iterations for each case proportion in datasets A1–A3 (vertical panels), analyzed with all differential relative abundance methods (horizontal panels). FPR is defined as the fraction of OTUs with *p* < 0.05. *P* values were not corrected for multiple testing. Black dots represent medians in each distribution. B. False positive rate distributions for dataset A4. Violin plot of distributions of false positive rate (FPR) in 150 iterations dataset A4, analyzed with all differential relative abundance methods (horizontal panels). FPR is defined as the fraction of OTUs with *p* < 0.05. *P* values were not corrected for multiple testing. Black dots represent medians in each distribution. (ZIP 649 kb)
Additional file 4: Figure S3. A. Area under the curve distributions for multiplicative spike-ins in dataset A1. Violin plot of distributions of area under the receiver operating characteristic curve (AUC) for spiked vs non-spiked *p* values from differential relative abundance (DA) tests. AUC distributions from 150 iterations for each combination of spike-in magnitude and case proportion (vertical panels) in dataset A1, analyzed with all differential relative abundance methods (horizontal panels). Black dots represent medians in each distribution. B. Area under the curve distributions for multiplicative spike-ins in dataset A2. Violin plot of distributions of area under the receiver operating characteristic curve (AUC) for spiked vs non-spiked *p* values from differential relative abundance (DA) tests. AUC distributions from 150 iterations for each combination of spike-in magnitude and case proportion (vertical panels) in dataset A2, analyzed with all differential relative abundance methods (horizontal panels). Black dots represent medians in each distribution. C. Area under the curve distributions for multiplicative spike-ins in dataset A3. Violin plot of distributions of area under the receiver operating characteristic curve (AUC) for spiked vs non-spiked *p* values from differential relative abundance (DA) tests. AUC distributions from 150 iterations for each combination of spike-in magnitude and case proportion (vertical panels) in dataset A3, analyzed with all differential relative abundance methods (horizontal panels). Black dots represent medians in each distribution. (ZIP 2685 kb)
Additional file 5: Figure S4. A. Area under the curve distributions for additive spike-ins in dataset A1. Violin plot of distributions of area under the receiver operating characteristic curve (AUC) for spiked vs non-spiked *p* values from differential relative abundance (DA) tests. AUC distributions from 150 iterations for each combination of spike-in magnitude and case proportion (vertical panels) in dataset A1, analyzed with all differential relative abundance methods (horizontal panels). Black dots represent medians in each distribution. B. Area under the curve distributions for additive spike-ins in dataset A2. Violin plot of distributions of area under the receiver operating characteristic curve (AUC) for spiked vs non-spiked *p* values from differential relative abundance (DA) tests. AUC distributions from 150 iterations for each combination of spike-in magnitude and case proportion (vertical panels) in dataset A2, analyzed with all differential relative abundance methods (horizontal panels). Black dots represent medians in each distribution. C. Area under the curve distributions for additive spike-ins in dataset A3. Violin plot of distributions of area under the receiver operating characteristic curve (AUC) for spiked vs non-spiked *p* values from differential relative abundance (DA) tests. AUC distributions from 150 iterations for each combination of spike-in magnitude and case proportion (vertical panels) in dataset A3, analyzed with all differential relative abundance methods (horizontal panels). Black dots represent medians in each distribution. (ZIP 2183 kb)
Additional file 6: Figure S5. Area under the curve distributions for mixed multiplicative spike-ins in datasets A1–A3. Violin plot of distributions of area under the receiver operating characteristic curve (AUC) for spiked vs non-spiked *p* values from differential relative abundance tests. AUC distributions from 150 iterations for each case proportion in datasets A1–A3 (vertical panels), analyzed with all differential relative abundance methods (horizontal panels). Black dots represent medians in each distribution. (PDF 588 kb)
Additional file 7: Figure S6. A. False positive rate distributions for datasets A1s–A3s and A1m–A3m. Violin plot of distributions of false positive rate (FPR) in 150 iterations for datasets A1s–A3s and A1m–A3m (vertical panels), analyzed with all differential relative abundance methods (horizontal panels). FPR is defined as the fraction of OTUs with *p* < 0.05. *P* values were not corrected for multiple testing. Black dots represent medians in each distribution. B. Area under the curve distributions for multiplicative spike-ins in datasets A1s–A3s and A1m–A3m. Violin plot of distributions of area under the receiver operating characteristic curve (AUC) for spiked vs non-spiked *p* values from differential relative abundance (DA) tests. AUC distributions from 150 iterations for each multiplicative spike-in magnitude in datasets A1s–A3s and A1m–A3m (vertical panels), analyzed with all differential relative abundance methods (horizontal panels). Black dots represent medians in each distribution. C. Area under the curve distributions for additive spike-ins in datasets A1s–A3s and A1m–A3m. Violin plot of distributions of area under the receiver operating characteristic curve (AUC) for spiked vs non-spiked *p* values from differential relative abundance (DA) tests. AUC distributions from 150 iterations for each additive spike-in magnitude in datasets A1s–A3s and A1m–A3m (vertical panels), analyzed with all differential relative abundance methods (horizontal panels). Black dots represent medians in each distribution. D. Area under the curve distributions for mixed multiplicative spike-ins in datasets A1s–A3s and A1m–A3m. Violin plot of distributions of area under the receiver operating characteristic curve (AUC) for spiked vs non-spiked *p* values from differential relative abundance (DA) tests. AUC distributions from 150 iterations for mixed multiplicative spike-in magnitudes in datasets A1s–A3s and A1m–A3m (vertical panels), analyzed with all differential relative abundance methods (horizontal panels). Black dots represent medians in each distribution. (ZIP 4027 kb)
Additional file 8: Figure S7. A. Area under the curve distributions for multiplicative spike-ins in dataset A4. Violin plot of distributions of area under the receiver operating characteristic curve (AUC) for spiked vs non-spiked *p* values from differential relative abundance (DA) tests. AUC distributions from 150 iterations for each combination of multiplicative spike-in magnitude and case proportion (vertical panels) in dataset A4, analyzed with all differential relative abundance methods (horizontal panels). Black dots represent medians in each distribution. B. Area under the curve distributions for additive spike-ins in dataset A4. Violin plot of distributions of area under the receiver operating characteristic curve (AUC) for spiked vs non-spiked *p* values from differential relative abundance (DA) tests. AUC distributions from 150 iterations for each combination of additive spike-in magnitude and case proportion (vertical panels) in dataset A4, analyzed with all differential relative abundance methods (horizontal panels). Black dots represent medians in each distribution. C. Area under the curve distributions for mixed multiplicative spike-ins in dataset A4. Violin plot of distributions of area under the receiver operating characteristic curve (AUC) for spiked vs non-spiked *p* values from differential relative abundance (DA) tests. AUC distributions from 150 iterations for each case proportion (vertical panels) in dataset A4, analyzed with all differential relative abundance methods (horizontal panels). Black dots represent medians in each distribution. (ZIP 1831 kb)
Additional file 9: Figure S8. A. Spike-in retrieval as a function of number of positive samples, by dataset size. Aggregated results across 150 iterations of multiplicative spike-ins of magnitude 5 with 50% cases, in datasets A1, A1s, and A1m. Each dot represents a spiked OTU. The Y-axis displays its *p* value quantile (0 is lowest *p* value, 1 is highest *p* value) within that DA run, and the X axis shows how many samples are positive (nonzero) for that OTU. Results from the three datasets are overlaid with different colors and faceted by statistical method. B. Spike-in retrieval as a function of number of positive samples, by case proportion. Aggregated results across 150 iterations of multiplicative spike-ins of magnitude 5 with 10, 25, or 50% cases, in dataset A1. Each dot represents a spiked OTU. The Y-axis displays its *p* value quantile (0 is lowest *p* value, 1 is highest *p* value) within that DA run, and the X axis shows how many samples are positive (nonzero) for that OTU. Results from the three case proportions are overlaid with different colors, and faceted by statistical method. C. Spike-in retrieval as a function of number of positive samples, by spike-in magnitude. Aggregated results across 150 iterations of multiplicative spike-ins of magnitudes 0.5, 2, 5, 10, and 20 with 50% cases, in dataset A1. Each dot represents a spiked OTU. The Y-axis displays its *p* value quantile (0 is lowest *p* value, 1 is highest *p* value) within that DA run, and the X axis shows how many samples are positive (nonzero) for that OTU. Results from the different spike-in magnitudes are overlaid with different colors, and faceted by statistical method. (ZIP 16398 kb)
Additional file 10: Figure S9. The effect of normalization, transformation, and distance metric on beta-diversity separation in the modified dataset B3. Dataset B3 modified by grouping of several OTUs, reducing the dataset to 8770 OTUs. All combinations of library size normalizations (horizontal panels) and count transformations (color) applied prior to calculation of distances and use of Adonis permanova model. Effect of age group measured as model *R* ^2^ value. The highest and lowest *R* ^2^ values (yielding best and worst separation, respectively) are demonstrated in subplots B & C as principal coordinate analysis plots, colored by design variable, with overlaid prediction ellipses. *CSS* cumulative sum scaling, *TMM* trimmed mean of M values, *TSS* total sum scaling. (PDF 124 kb)
Additional file 11: Table S3. Summary of benchmark performance for included tools. (PDF 32 kb)
Additional file 12: Figure S10. The lowest obtainable *p* values from a Wilcoxon rank sum test depend on sample size and sparsity. Overview of the lowest *p* value theoretically obtainable using optimal conditions for a given sample size and level of sparsity. Optimal conditions refer to (a) 50% cases, i.e., *n*1 = *n*2, (b) only nonzero counts in one of the groups, limited by level of sparsity, (c) no ties between nonzero counts. One sided *p* values from normal approximation shown. Since the *p* value only depends on the rank distribution, the actual count values do not matter. Color of the lines indicates level of sparsity; dotted red line corresponds to *p* = 0.05. (PDF 9 kb)
Additional file 13: Supplementary methods. (DOCX 15 kb)
Additional file 14: Table S2. Chimeras removed from dataset B3. (XLSX 49 kb)

## Abbreviations

AUC: Area under the receiver operating characteristic (ROC) curve
CSS: Cumulative sum scaling
DA: Differential (relative) abundance
FPR: False positive rate
GLM: Generalized linear model
OTU: Operational taxonomic unit
PCoA: Principal coordinates analysis
ROC: Receiver operating characteristic
TMM: Trimmed mean of *M* values
