# Supplementary figures and images for: Large-scale benchmarking reveals false discoveries and count transformation sensitivity in 16S rRNA gene amplicon data analysis methods used in microbiome studies
Source: Microbiome. 2016 Nov 25;4:62. doi: 10.1186/s40168-016-0208-8 (PMC5123278; doi:10.1186/s40168-016-0208-8)

**A**

2153 OTUs

622 Samples

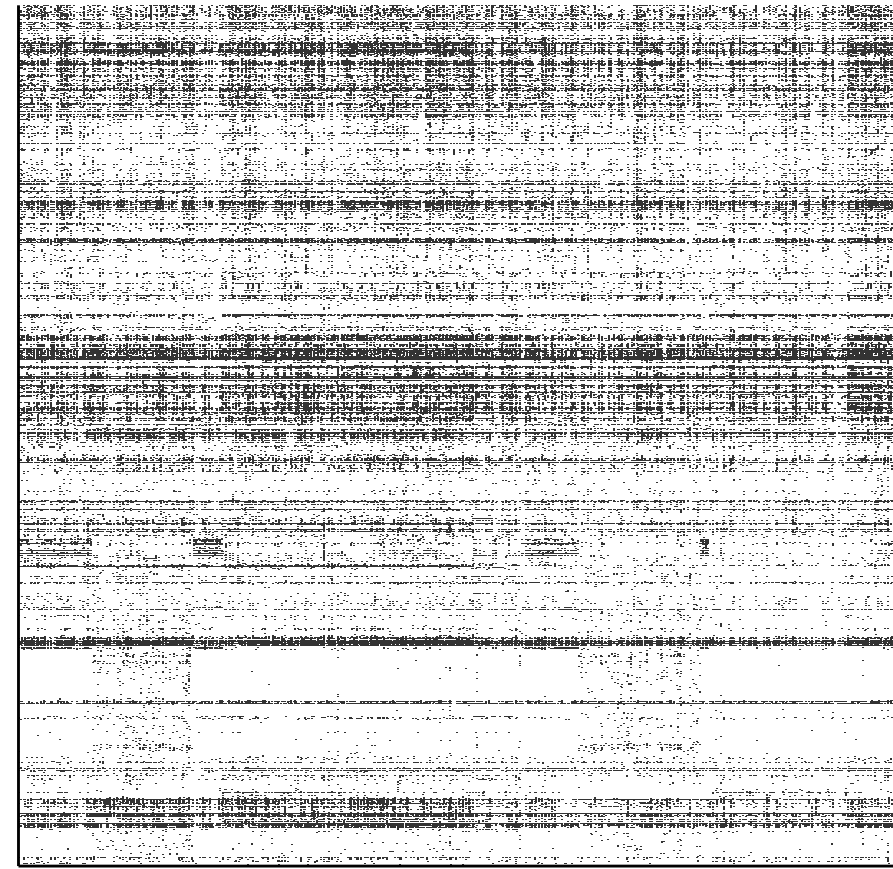**B**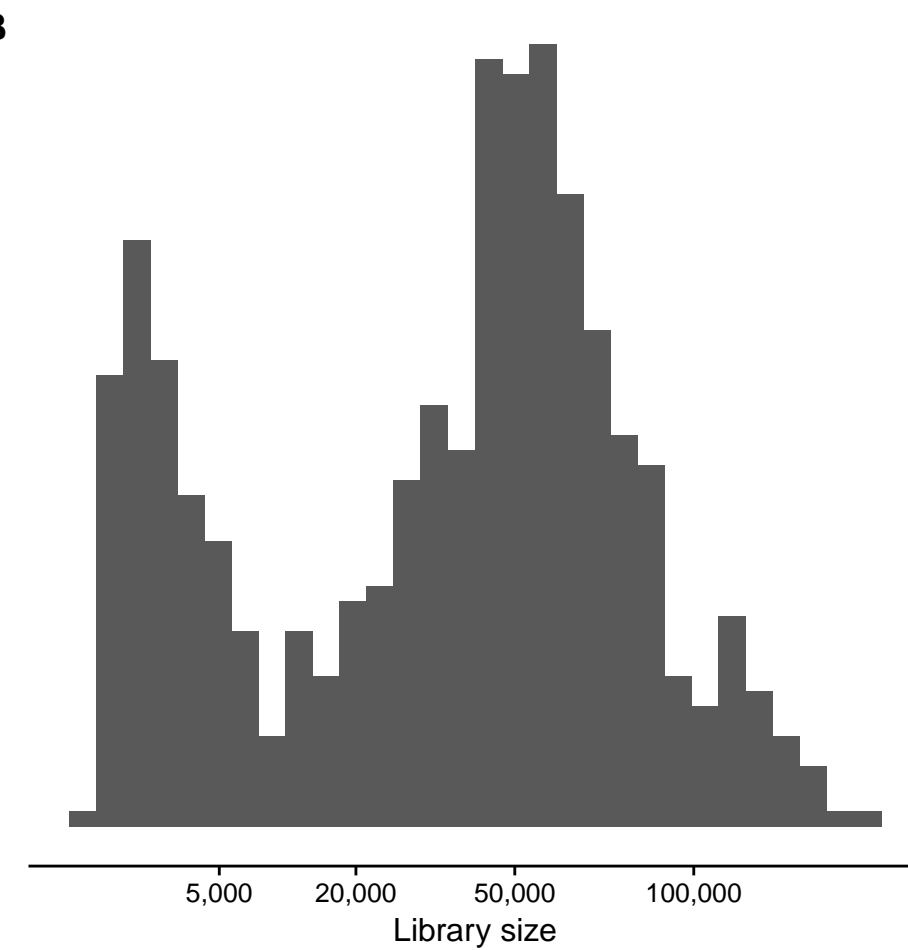**C**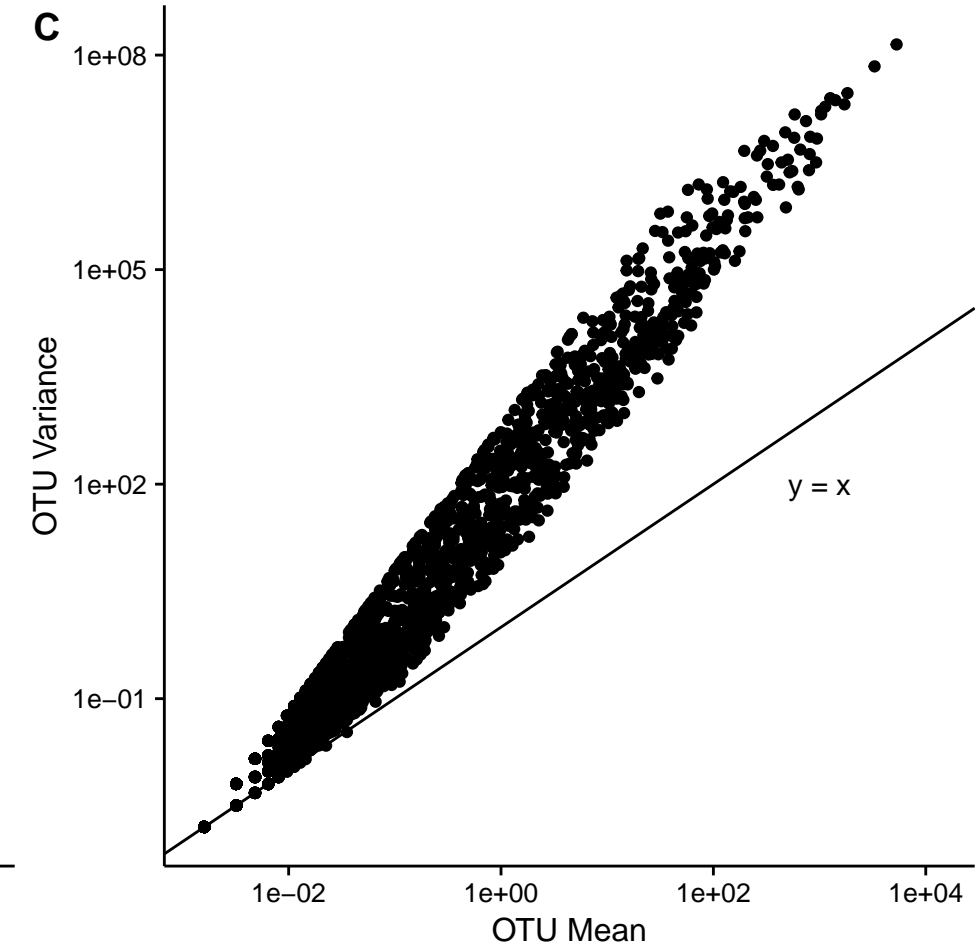

Supplement: Additional file 2: Figure S1. — Data characteristics for feces dataset A1. (A) Dot plot overview of the dataset; black if an OTU was present, blank if not present in a given sample. Sparsity in the dataset is 90.8%. (B) Library size distribution showing differences of several orders of magnitude. (C) Mean-variance relationship showing overdispersion, i.e., higher variance than mean value of a given OTU. (PDF 504 kb) [file 40168_2016_208_MOESM2_ESM.pdf]

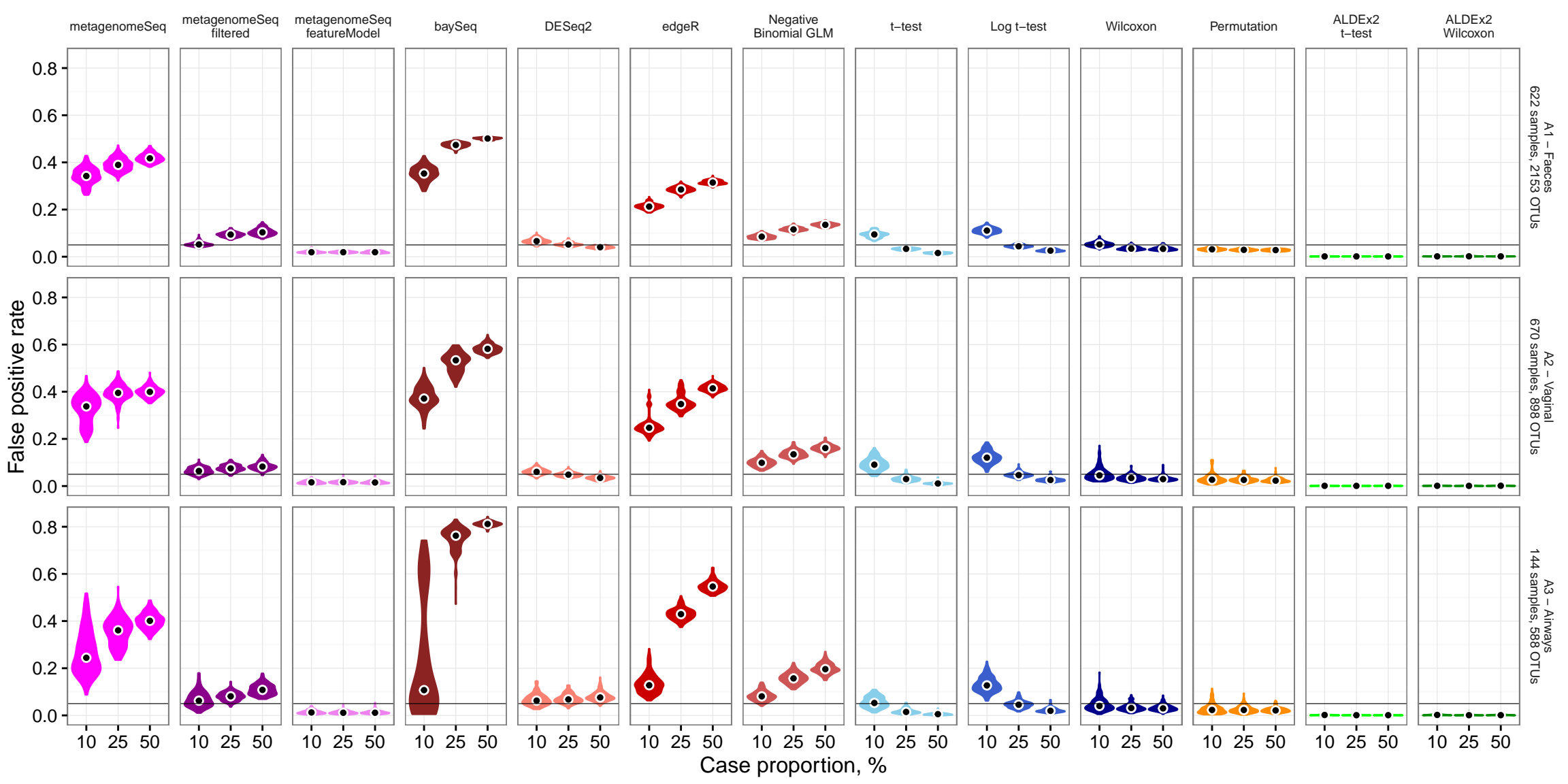

Supplement: Additional file 3: Figure S2. — A. False positive rate distributions for datasets A1–A3. Violin plot of distributions of false positive rate (FPR) in 150 iterations for each case proportion in datasets A1–A3 (vertical panels), analyzed with all differential relative abundance methods (horizontal panels). FPR is defined as the fraction of OTUs with p < 0.05. P values were not corrected for multiple testing. Black dots represent medians in each distribution. B. False positive rate distributions for dataset A4. Violin plot of distributions of false positive rate (FPR) in 150 iterations dataset A4, analyzed with all differential relative abundance methods (horizontal panels). FPR is defined as the fraction of OTUs with p < 0.05. P values were not corrected for multiple testing. Black dots represent medians in each distribution. (ZIP 649 kb) [file 40168_2016_208_MOESM3_ESM.zip › S2A_Figure.pdf]

False positive rate

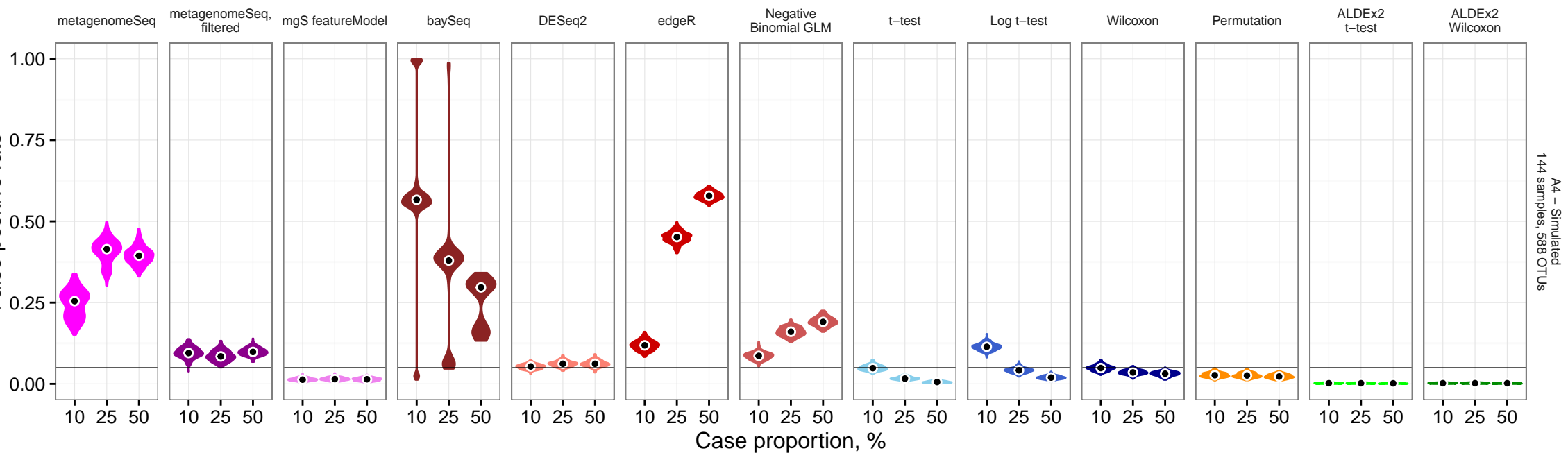

Supplement: Additional file 3: Figure S2. — A. False positive rate distributions for datasets A1–A3. Violin plot of distributions of false positive rate (FPR) in 150 iterations for each case proportion in datasets A1–A3 (vertical panels), analyzed with all differential relative abundance methods (horizontal panels). FPR is defined as the fraction of OTUs with p < 0.05. P values were not corrected for multiple testing. Black dots represent medians in each distribution. B. False positive rate distributions for dataset A4. Violin plot of distributions of false positive rate (FPR) in 150 iterations dataset A4, analyzed with all differential relative abundance methods (horizontal panels). FPR is defined as the fraction of OTUs with p < 0.05. P values were not corrected for multiple testing. Black dots represent medians in each distribution. (ZIP 649 kb) [file 40168_2016_208_MOESM3_ESM.zip › S2B_Figure.pdf]

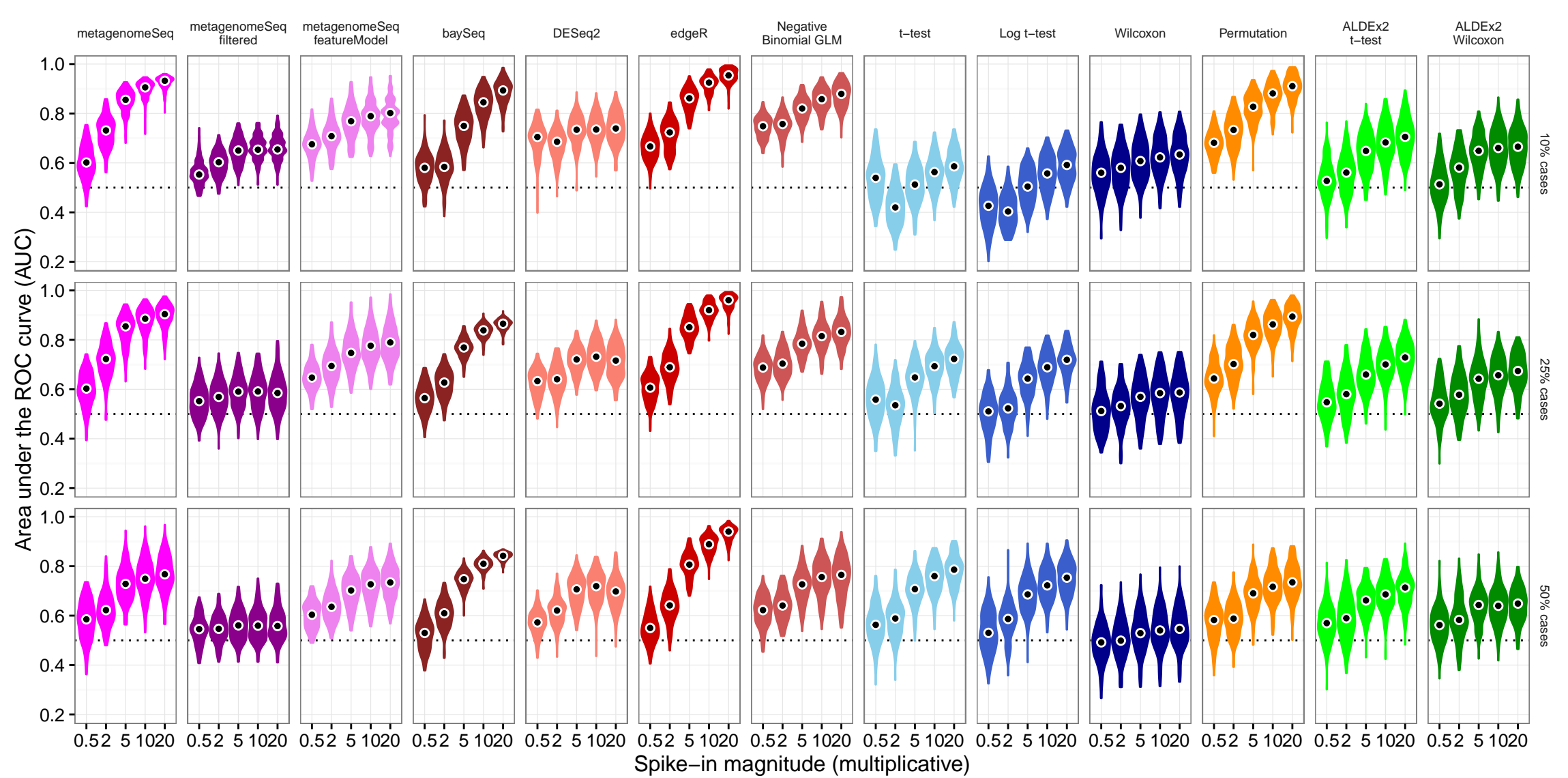

Supplement: Additional file 4: Figure S3. — A. Area under the curve distributions for multiplicative spike-ins in dataset A1. Violin plot of distributions of area under the receiver operating characteristic curve (AUC) for spiked vs non-spiked p values from differential relative abundance (DA) tests. AUC distributions from 150 iterations for each combination of spike-in magnitude and case proportion (vertical panels) in dataset A1, analyzed with all differential relative abundance methods (horizontal panels). Black dots represent medians in each distribution. B. Area under the curve distributions for multiplicative spike-ins in dataset A2. Violin plot of distributions of area under the receiver operating characteristic curve (AUC) for spiked vs non-spiked p values from differential relative abundance (DA) tests. AUC distributions from 150 iterations for each combination of spike-in magnitude and case proportion (vertical panels) in dataset A2, analyzed with all differential relative abundance methods (horizontal panels). Black dots represent medians in each distribution. C. Area under the curve distributions for multiplicative spike-ins in dataset A3. Violin plot of distributions of area under the receiver operating characteristic curve (AUC) for spiked vs non-spiked p values from differential relative abundance (DA) tests. AUC distributions from 150 iterations for each combination of spike-in magnitude and case proportion (vertical panels) in dataset A3, analyzed with all differential relative abundance methods (horizontal panels). Black dots represent medians in each distribution. (ZIP 2685 kb) [file 40168_2016_208_MOESM4_ESM.zip › S3A_Figure.pdf]

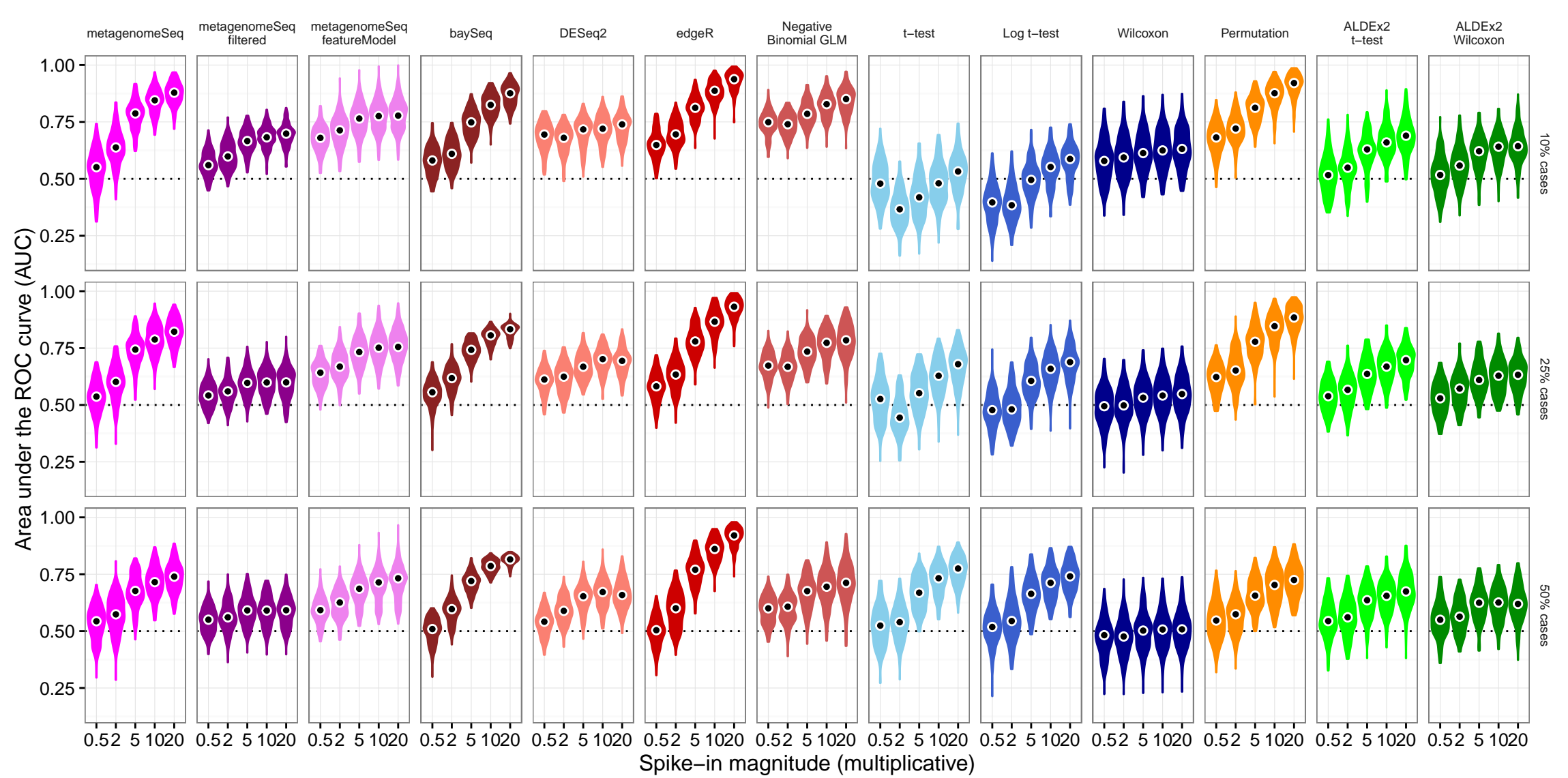

Supplement: Additional file 4: Figure S3. — A. Area under the curve distributions for multiplicative spike-ins in dataset A1. Violin plot of distributions of area under the receiver operating characteristic curve (AUC) for spiked vs non-spiked p values from differential relative abundance (DA) tests. AUC distributions from 150 iterations for each combination of spike-in magnitude and case proportion (vertical panels) in dataset A1, analyzed with all differential relative abundance methods (horizontal panels). Black dots represent medians in each distribution. B. Area under the curve distributions for multiplicative spike-ins in dataset A2. Violin plot of distributions of area under the receiver operating characteristic curve (AUC) for spiked vs non-spiked p values from differential relative abundance (DA) tests. AUC distributions from 150 iterations for each combination of spike-in magnitude and case proportion (vertical panels) in dataset A2, analyzed with all differential relative abundance methods (horizontal panels). Black dots represent medians in each distribution. C. Area under the curve distributions for multiplicative spike-ins in dataset A3. Violin plot of distributions of area under the receiver operating characteristic curve (AUC) for spiked vs non-spiked p values from differential relative abundance (DA) tests. AUC distributions from 150 iterations for each combination of spike-in magnitude and case proportion (vertical panels) in dataset A3, analyzed with all differential relative abundance methods (horizontal panels). Black dots represent medians in each distribution. (ZIP 2685 kb) [file 40168_2016_208_MOESM4_ESM.zip › S3B_Figure.pdf]

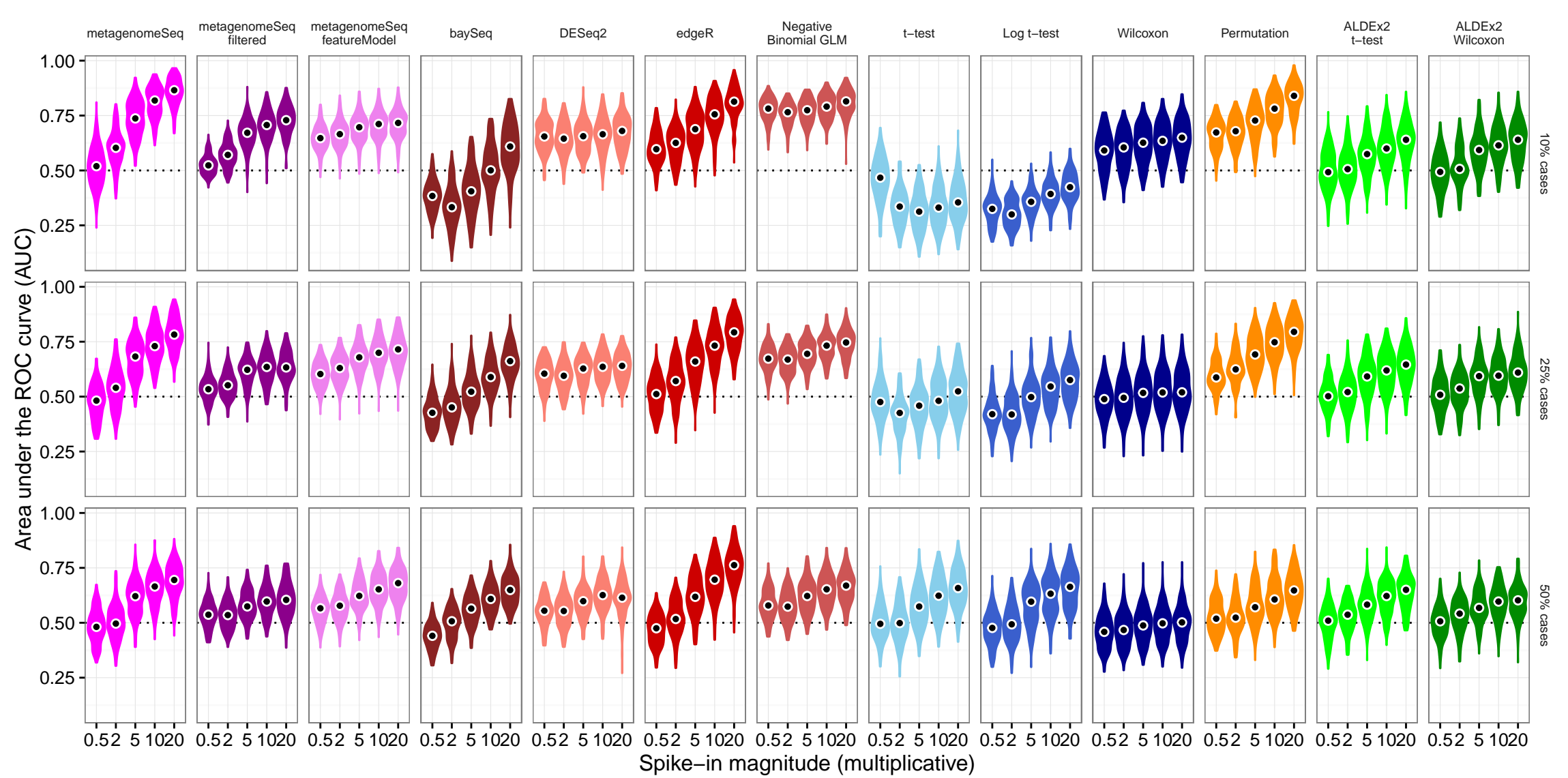

Supplement: Additional file 4: Figure S3. — A. Area under the curve distributions for multiplicative spike-ins in dataset A1. Violin plot of distributions of area under the receiver operating characteristic curve (AUC) for spiked vs non-spiked p values from differential relative abundance (DA) tests. AUC distributions from 150 iterations for each combination of spike-in magnitude and case proportion (vertical panels) in dataset A1, analyzed with all differential relative abundance methods (horizontal panels). Black dots represent medians in each distribution. B. Area under the curve distributions for multiplicative spike-ins in dataset A2. Violin plot of distributions of area under the receiver operating characteristic curve (AUC) for spiked vs non-spiked p values from differential relative abundance (DA) tests. AUC distributions from 150 iterations for each combination of spike-in magnitude and case proportion (vertical panels) in dataset A2, analyzed with all differential relative abundance methods (horizontal panels). Black dots represent medians in each distribution. C. Area under the curve distributions for multiplicative spike-ins in dataset A3. Violin plot of distributions of area under the receiver operating characteristic curve (AUC) for spiked vs non-spiked p values from differential relative abundance (DA) tests. AUC distributions from 150 iterations for each combination of spike-in magnitude and case proportion (vertical panels) in dataset A3, analyzed with all differential relative abundance methods (horizontal panels). Black dots represent medians in each distribution. (ZIP 2685 kb) [file 40168_2016_208_MOESM4_ESM.zip › S3C_Figure.pdf]

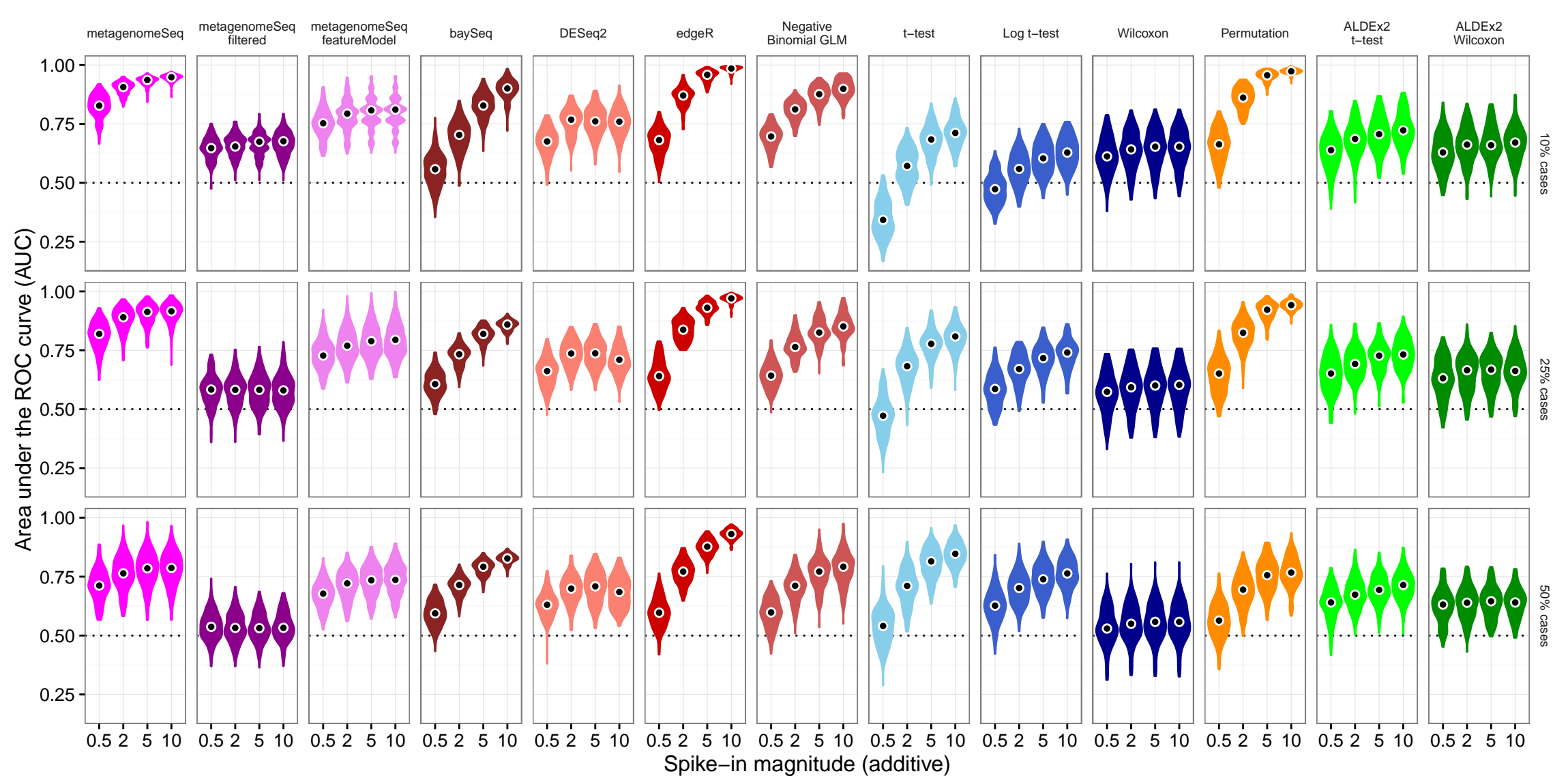

Supplement: Additional file 5: Figure S4. — A. Area under the curve distributions for additive spike-ins in dataset A1. Violin plot of distributions of area under the receiver operating characteristic curve (AUC) for spiked vs non-spiked p values from differential relative abundance (DA) tests. AUC distributions from 150 iterations for each combination of spike-in magnitude and case proportion (vertical panels) in dataset A1, analyzed with all differential relative abundance methods (horizontal panels). Black dots represent medians in each distribution. B. Area under the curve distributions for additive spike-ins in dataset A2. Violin plot of distributions of area under the receiver operating characteristic curve (AUC) for spiked vs non-spiked p values from differential relative abundance (DA) tests. AUC distributions from 150 iterations for each combination of spike-in magnitude and case proportion (vertical panels) in dataset A2, analyzed with all differential relative abundance methods (horizontal panels). Black dots represent medians in each distribution. C. Area under the curve distributions for additive spike-ins in dataset A3. Violin plot of distributions of area under the receiver operating characteristic curve (AUC) for spiked vs non-spiked p values from differential relative abundance (DA) tests. AUC distributions from 150 iterations for each combination of spike-in magnitude and case proportion (vertical panels) in dataset A3, analyzed with all differential relative abundance methods (horizontal panels). Black dots represent medians in each distribution. (ZIP 2183 kb) [file 40168_2016_208_MOESM5_ESM.zip › S4A_Figure.pdf]

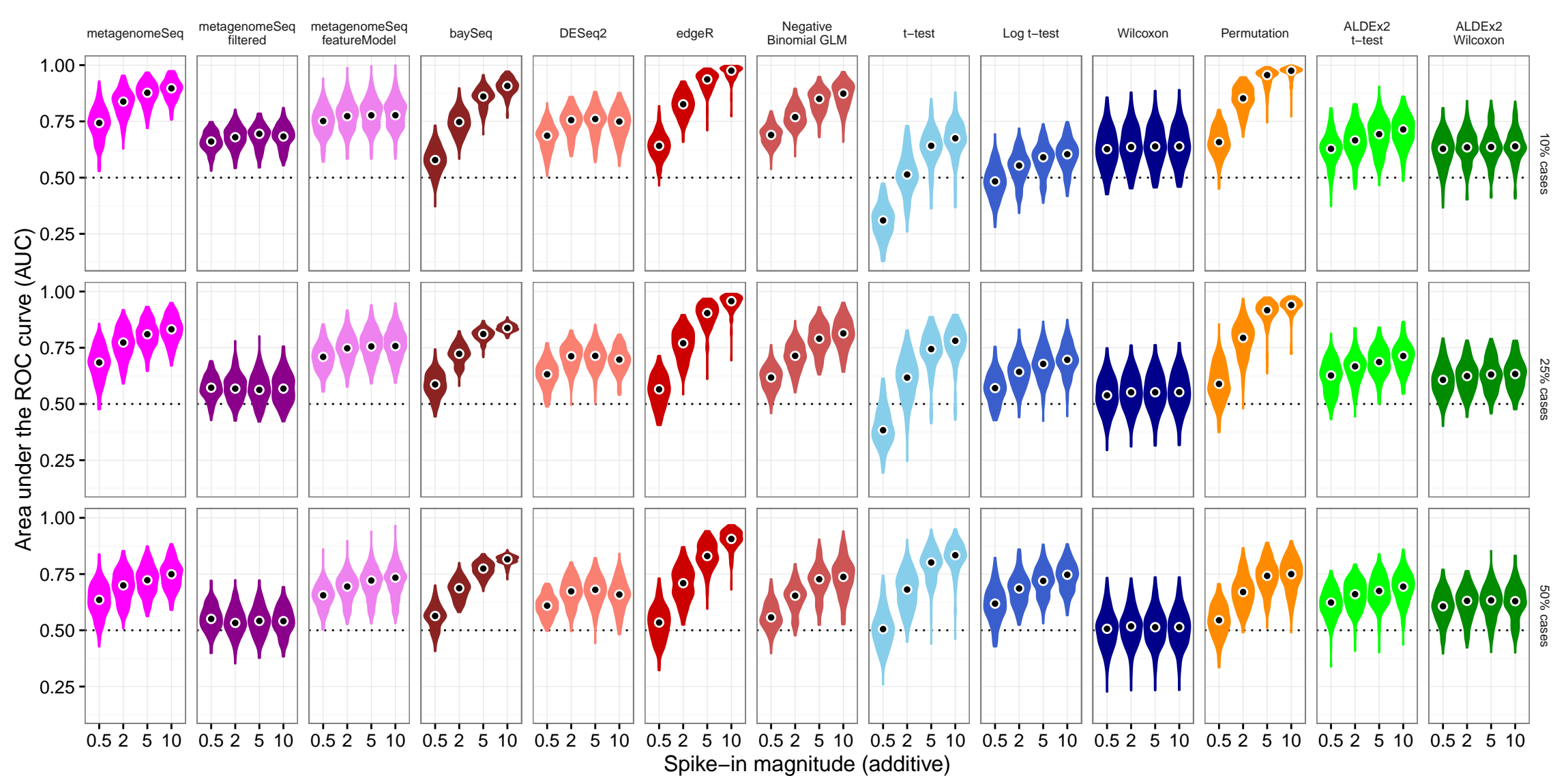

Supplement: Additional file 5: Figure S4. — A. Area under the curve distributions for additive spike-ins in dataset A1. Violin plot of distributions of area under the receiver operating characteristic curve (AUC) for spiked vs non-spiked p values from differential relative abundance (DA) tests. AUC distributions from 150 iterations for each combination of spike-in magnitude and case proportion (vertical panels) in dataset A1, analyzed with all differential relative abundance methods (horizontal panels). Black dots represent medians in each distribution. B. Area under the curve distributions for additive spike-ins in dataset A2. Violin plot of distributions of area under the receiver operating characteristic curve (AUC) for spiked vs non-spiked p values from differential relative abundance (DA) tests. AUC distributions from 150 iterations for each combination of spike-in magnitude and case proportion (vertical panels) in dataset A2, analyzed with all differential relative abundance methods (horizontal panels). Black dots represent medians in each distribution. C. Area under the curve distributions for additive spike-ins in dataset A3. Violin plot of distributions of area under the receiver operating characteristic curve (AUC) for spiked vs non-spiked p values from differential relative abundance (DA) tests. AUC distributions from 150 iterations for each combination of spike-in magnitude and case proportion (vertical panels) in dataset A3, analyzed with all differential relative abundance methods (horizontal panels). Black dots represent medians in each distribution. (ZIP 2183 kb) [file 40168_2016_208_MOESM5_ESM.zip › S4B_Figure.pdf]

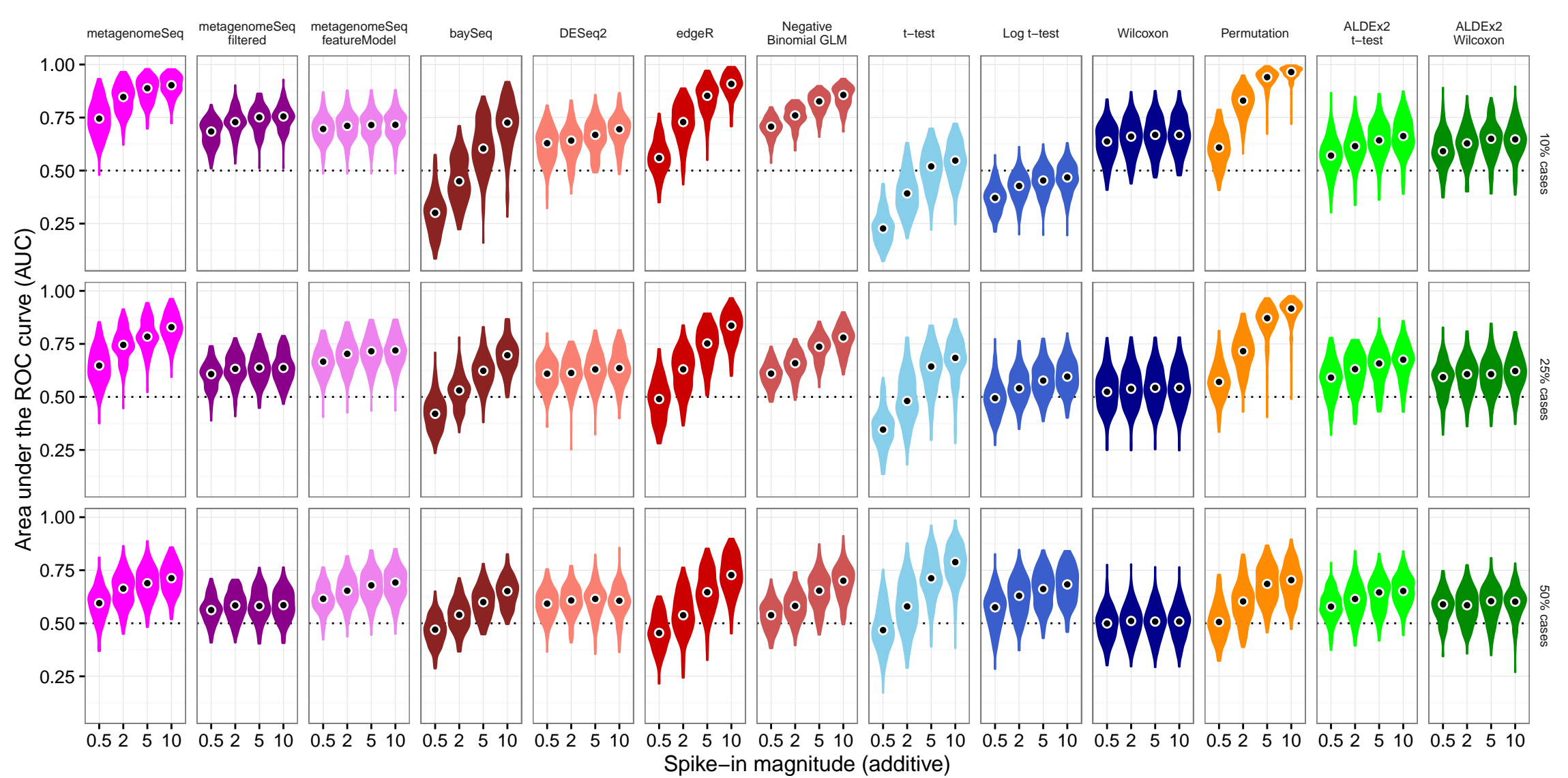

Supplement: Additional file 5: Figure S4. — A. Area under the curve distributions for additive spike-ins in dataset A1. Violin plot of distributions of area under the receiver operating characteristic curve (AUC) for spiked vs non-spiked p values from differential relative abundance (DA) tests. AUC distributions from 150 iterations for each combination of spike-in magnitude and case proportion (vertical panels) in dataset A1, analyzed with all differential relative abundance methods (horizontal panels). Black dots represent medians in each distribution. B. Area under the curve distributions for additive spike-ins in dataset A2. Violin plot of distributions of area under the receiver operating characteristic curve (AUC) for spiked vs non-spiked p values from differential relative abundance (DA) tests. AUC distributions from 150 iterations for each combination of spike-in magnitude and case proportion (vertical panels) in dataset A2, analyzed with all differential relative abundance methods (horizontal panels). Black dots represent medians in each distribution. C. Area under the curve distributions for additive spike-ins in dataset A3. Violin plot of distributions of area under the receiver operating characteristic curve (AUC) for spiked vs non-spiked p values from differential relative abundance (DA) tests. AUC distributions from 150 iterations for each combination of spike-in magnitude and case proportion (vertical panels) in dataset A3, analyzed with all differential relative abundance methods (horizontal panels). Black dots represent medians in each distribution. (ZIP 2183 kb) [file 40168_2016_208_MOESM5_ESM.zip › S4C_Figure.pdf]

Area under the ROC curve (AUC),  
mixed multiplicative spiking

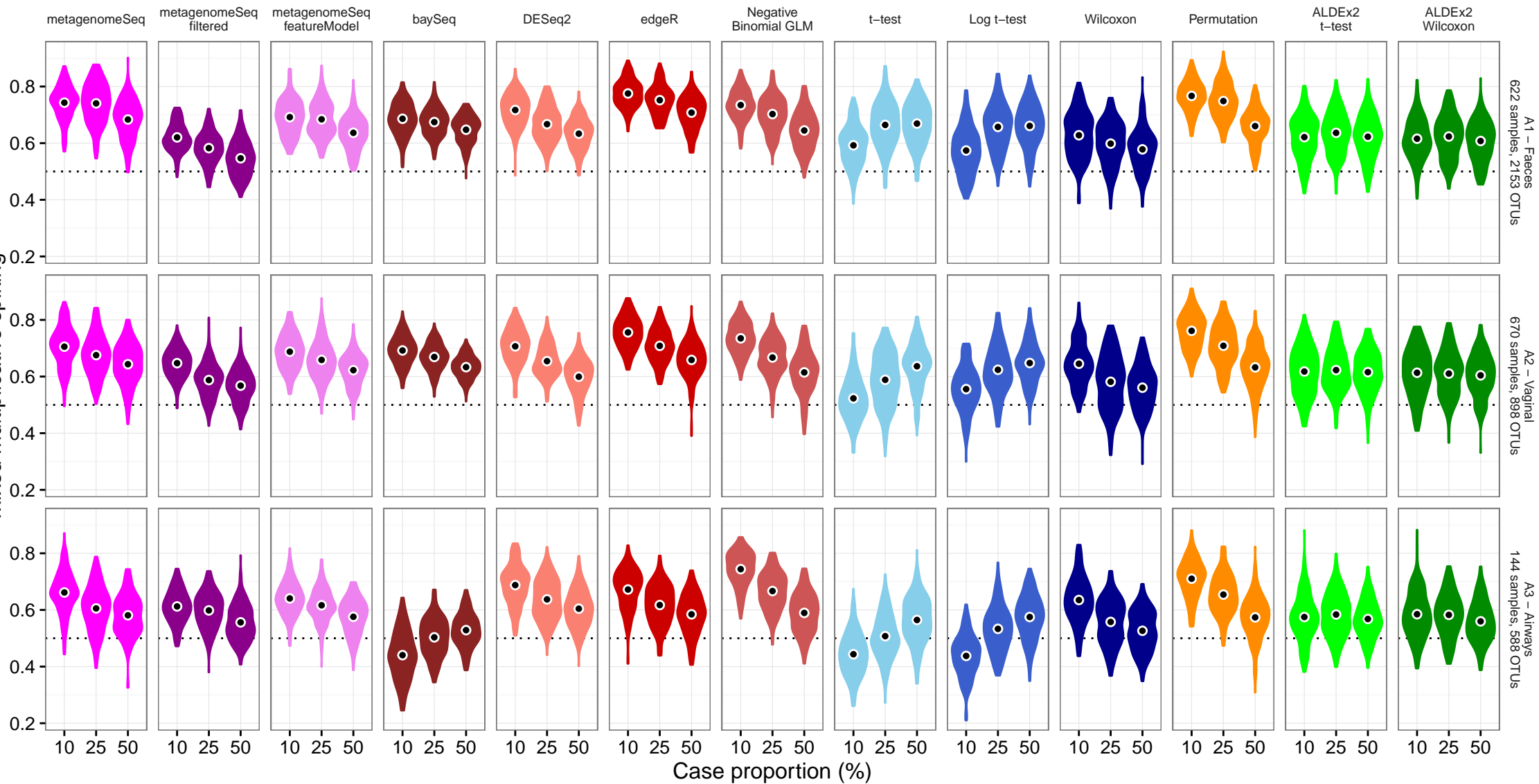

Supplement: Additional file 6: Figure S5. — Area under the curve distributions for mixed multiplicative spike-ins in datasets A1–A3. Violin plot of distributions of area under the receiver operating characteristic curve (AUC) for spiked vs non-spiked p values from differential relative abundance tests. AUC distributions from 150 iterations for each case proportion in datasets A1–A3 (vertical panels), analyzed with all differential relative abundance methods (horizontal panels). Black dots represent medians in each distribution. (PDF 588 kb) [file 40168_2016_208_MOESM6_ESM.pdf]

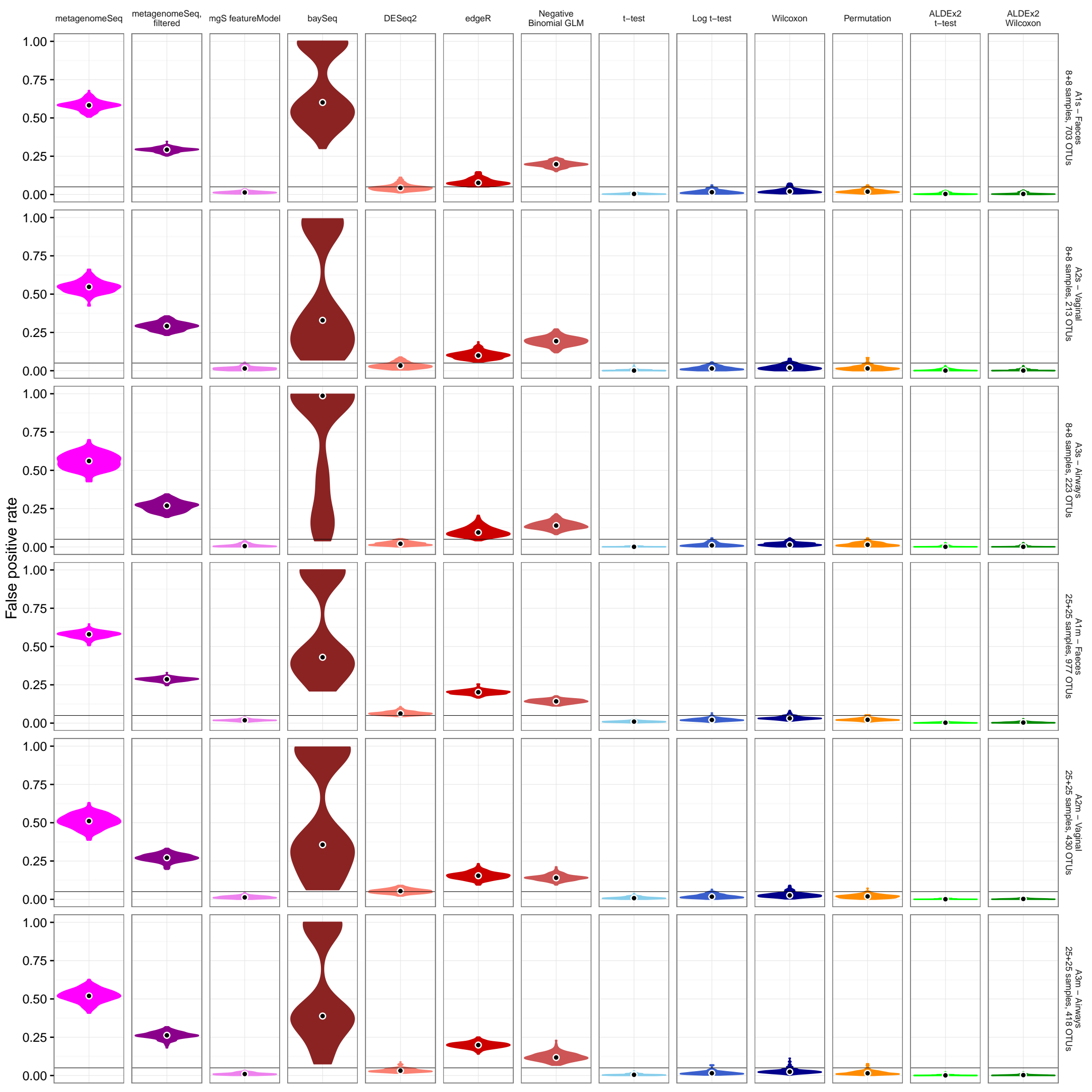

Supplement: Additional file 7: Figure S6. — A. False positive rate distributions for datasets A1s–A3s and A1m–A3m. Violin plot of distributions of false positive rate (FPR) in 150 iterations for datasets A1s–A3s and A1m–A3m (vertical panels), analyzed with all differential relative abundance methods (horizontal panels). FPR is defined as the fraction of OTUs with p < 0.05. P values were not corrected for multiple testing. Black dots represent medians in each distribution. B. Area under the curve distributions for multiplicative spike-ins in datasets A1s–A3s and A1m–A3m. Violin plot of distributions of area under the receiver operating characteristic curve (AUC) for spiked vs non-spiked p values from differential relative abundance (DA) tests. AUC distributions from 150 iterations for each multiplicative spike-in magnitude in datasets A1s–A3s and A1m–A3m (vertical panels), analyzed with all differential relative abundance methods (horizontal panels). Black dots represent medians in each distribution. C. Area under the curve distributions for additive spike-ins in datasets A1s–A3s and A1m–A3m. Violin plot of distributions of area under the receiver operating characteristic curve (AUC) for spiked vs non-spiked p values from differential relative abundance (DA) tests. AUC distributions from 150 iterations for each additive spike-in magnitude in datasets A1s–A3s and A1m–A3m (vertical panels), analyzed with all differential relative abundance methods (horizontal panels). Black dots represent medians in each distribution. D. Area under the curve distributions for mixed multiplicative spike-ins in datasets A1s–A3s and A1m–A3m. Violin plot of distributions of area under the receiver operating characteristic curve (AUC) for spiked vs non-spiked p values from differential relative abundance (DA) tests. AUC distributions from 150 iterations for mixed multiplicative spike-in magnitudes in datasets A1s–A3s and A1m–A3m (vertical panels), analyzed with all differential relative abundance methods (horizontal panels). Black do [file 40168_2016_208_MOESM7_ESM.zip › S6A_Figure.pdf]

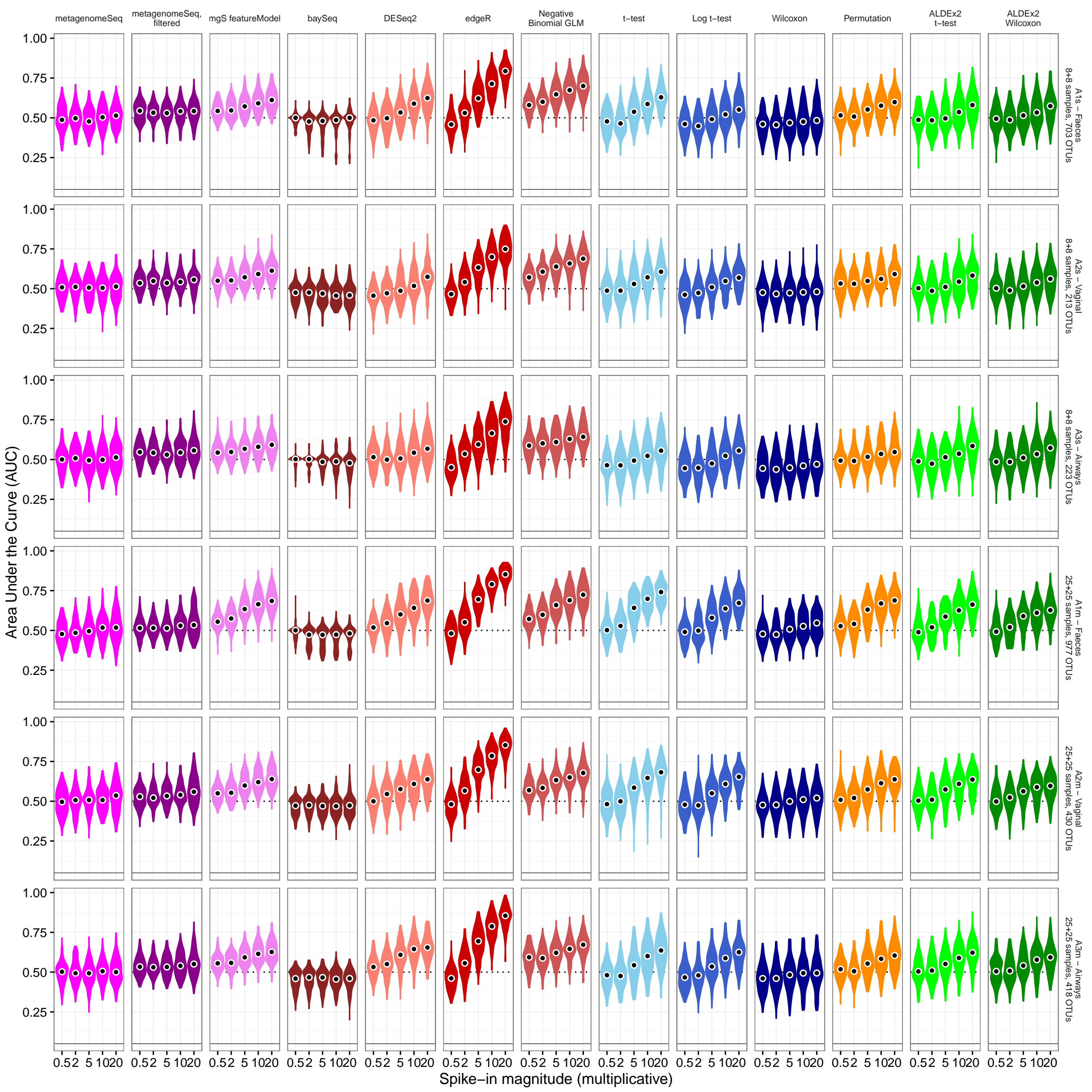

Supplement: Additional file 7: Figure S6. — A. False positive rate distributions for datasets A1s–A3s and A1m–A3m. Violin plot of distributions of false positive rate (FPR) in 150 iterations for datasets A1s–A3s and A1m–A3m (vertical panels), analyzed with all differential relative abundance methods (horizontal panels). FPR is defined as the fraction of OTUs with p < 0.05. P values were not corrected for multiple testing. Black dots represent medians in each distribution. B. Area under the curve distributions for multiplicative spike-ins in datasets A1s–A3s and A1m–A3m. Violin plot of distributions of area under the receiver operating characteristic curve (AUC) for spiked vs non-spiked p values from differential relative abundance (DA) tests. AUC distributions from 150 iterations for each multiplicative spike-in magnitude in datasets A1s–A3s and A1m–A3m (vertical panels), analyzed with all differential relative abundance methods (horizontal panels). Black dots represent medians in each distribution. C. Area under the curve distributions for additive spike-ins in datasets A1s–A3s and A1m–A3m. Violin plot of distributions of area under the receiver operating characteristic curve (AUC) for spiked vs non-spiked p values from differential relative abundance (DA) tests. AUC distributions from 150 iterations for each additive spike-in magnitude in datasets A1s–A3s and A1m–A3m (vertical panels), analyzed with all differential relative abundance methods (horizontal panels). Black dots represent medians in each distribution. D. Area under the curve distributions for mixed multiplicative spike-ins in datasets A1s–A3s and A1m–A3m. Violin plot of distributions of area under the receiver operating characteristic curve (AUC) for spiked vs non-spiked p values from differential relative abundance (DA) tests. AUC distributions from 150 iterations for mixed multiplicative spike-in magnitudes in datasets A1s–A3s and A1m–A3m (vertical panels), analyzed with all differential relative abundance methods (horizontal panels). Black do [file 40168_2016_208_MOESM7_ESM.zip › S6B_Figure.pdf]

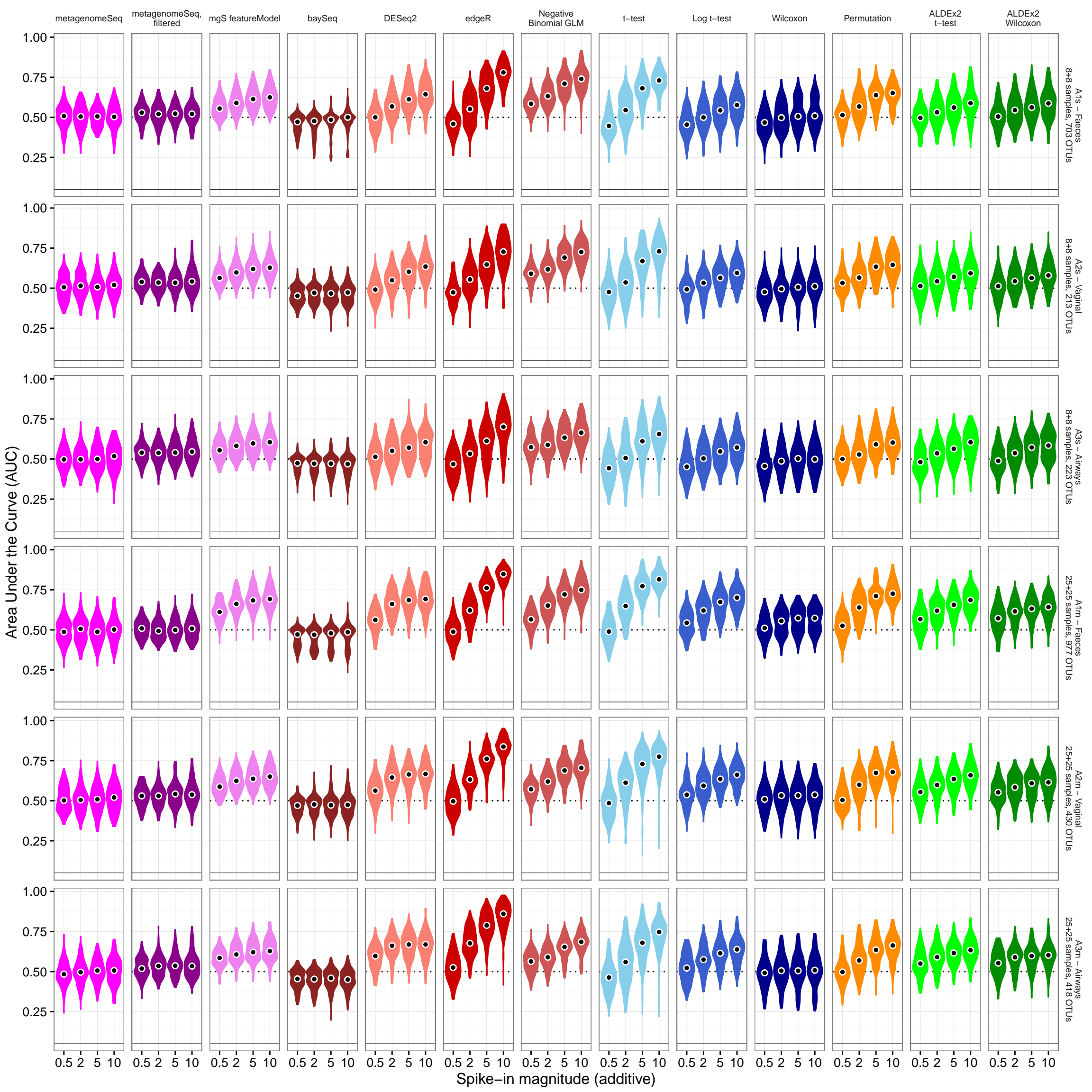

Supplement: Additional file 7: Figure S6. — A. False positive rate distributions for datasets A1s–A3s and A1m–A3m. Violin plot of distributions of false positive rate (FPR) in 150 iterations for datasets A1s–A3s and A1m–A3m (vertical panels), analyzed with all differential relative abundance methods (horizontal panels). FPR is defined as the fraction of OTUs with p < 0.05. P values were not corrected for multiple testing. Black dots represent medians in each distribution. B. Area under the curve distributions for multiplicative spike-ins in datasets A1s–A3s and A1m–A3m. Violin plot of distributions of area under the receiver operating characteristic curve (AUC) for spiked vs non-spiked p values from differential relative abundance (DA) tests. AUC distributions from 150 iterations for each multiplicative spike-in magnitude in datasets A1s–A3s and A1m–A3m (vertical panels), analyzed with all differential relative abundance methods (horizontal panels). Black dots represent medians in each distribution. C. Area under the curve distributions for additive spike-ins in datasets A1s–A3s and A1m–A3m. Violin plot of distributions of area under the receiver operating characteristic curve (AUC) for spiked vs non-spiked p values from differential relative abundance (DA) tests. AUC distributions from 150 iterations for each additive spike-in magnitude in datasets A1s–A3s and A1m–A3m (vertical panels), analyzed with all differential relative abundance methods (horizontal panels). Black dots represent medians in each distribution. D. Area under the curve distributions for mixed multiplicative spike-ins in datasets A1s–A3s and A1m–A3m. Violin plot of distributions of area under the receiver operating characteristic curve (AUC) for spiked vs non-spiked p values from differential relative abundance (DA) tests. AUC distributions from 150 iterations for mixed multiplicative spike-in magnitudes in datasets A1s–A3s and A1m–A3m (vertical panels), analyzed with all differential relative abundance methods (horizontal panels). Black do [file 40168_2016_208_MOESM7_ESM.zip › S6C_Figure.pdf]

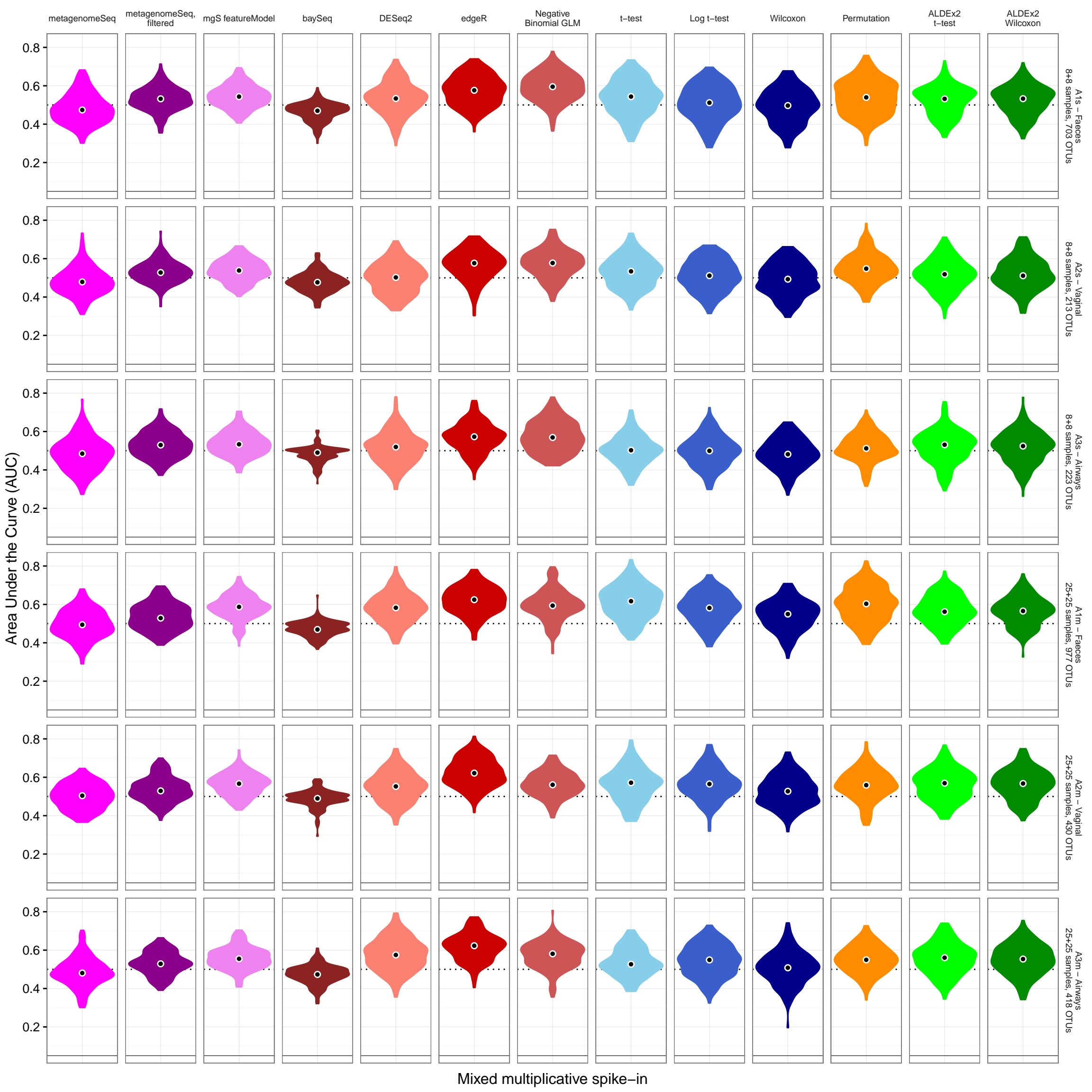

Supplement: Additional file 7: Figure S6. — A. False positive rate distributions for datasets A1s–A3s and A1m–A3m. Violin plot of distributions of false positive rate (FPR) in 150 iterations for datasets A1s–A3s and A1m–A3m (vertical panels), analyzed with all differential relative abundance methods (horizontal panels). FPR is defined as the fraction of OTUs with p < 0.05. P values were not corrected for multiple testing. Black dots represent medians in each distribution. B. Area under the curve distributions for multiplicative spike-ins in datasets A1s–A3s and A1m–A3m. Violin plot of distributions of area under the receiver operating characteristic curve (AUC) for spiked vs non-spiked p values from differential relative abundance (DA) tests. AUC distributions from 150 iterations for each multiplicative spike-in magnitude in datasets A1s–A3s and A1m–A3m (vertical panels), analyzed with all differential relative abundance methods (horizontal panels). Black dots represent medians in each distribution. C. Area under the curve distributions for additive spike-ins in datasets A1s–A3s and A1m–A3m. Violin plot of distributions of area under the receiver operating characteristic curve (AUC) for spiked vs non-spiked p values from differential relative abundance (DA) tests. AUC distributions from 150 iterations for each additive spike-in magnitude in datasets A1s–A3s and A1m–A3m (vertical panels), analyzed with all differential relative abundance methods (horizontal panels). Black dots represent medians in each distribution. D. Area under the curve distributions for mixed multiplicative spike-ins in datasets A1s–A3s and A1m–A3m. Violin plot of distributions of area under the receiver operating characteristic curve (AUC) for spiked vs non-spiked p values from differential relative abundance (DA) tests. AUC distributions from 150 iterations for mixed multiplicative spike-in magnitudes in datasets A1s–A3s and A1m–A3m (vertical panels), analyzed with all differential relative abundance methods (horizontal panels). Black do [file 40168_2016_208_MOESM7_ESM.zip › S6D_Figure.pdf]

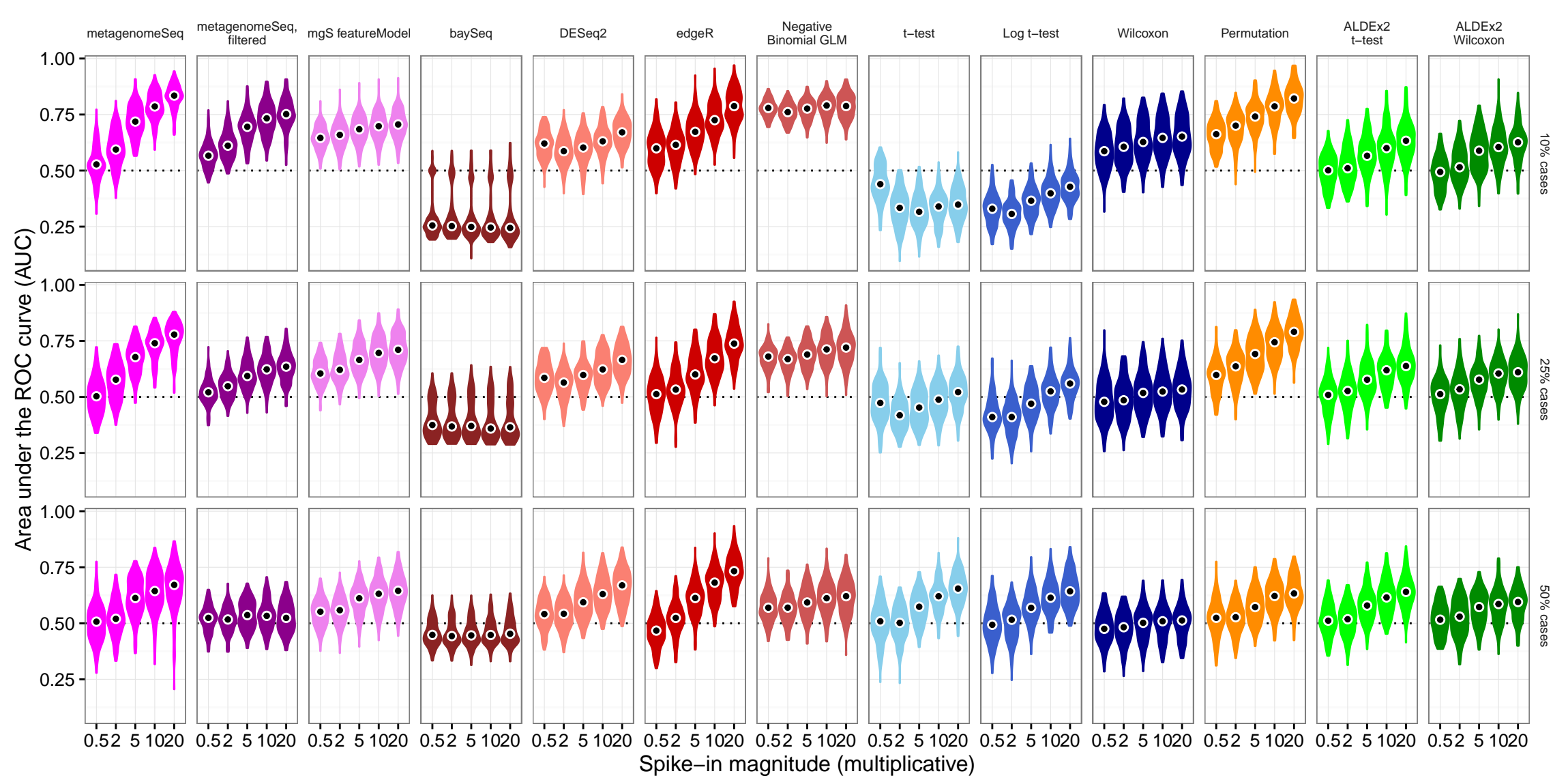

Supplement: Additional file 8: Figure S7. — A. Area under the curve distributions for multiplicative spike-ins in dataset A4. Violin plot of distributions of area under the receiver operating characteristic curve (AUC) for spiked vs non-spiked p values from differential relative abundance (DA) tests. AUC distributions from 150 iterations for each combination of multiplicative spike-in magnitude and case proportion (vertical panels) in dataset A4, analyzed with all differential relative abundance methods (horizontal panels). Black dots represent medians in each distribution. B. Area under the curve distributions for additive spike-ins in dataset A4. Violin plot of distributions of area under the receiver operating characteristic curve (AUC) for spiked vs non-spiked p values from differential relative abundance (DA) tests. AUC distributions from 150 iterations for each combination of additive spike-in magnitude and case proportion (vertical panels) in dataset A4, analyzed with all differential relative abundance methods (horizontal panels). Black dots represent medians in each distribution. C. Area under the curve distributions for mixed multiplicative spike-ins in dataset A4. Violin plot of distributions of area under the receiver operating characteristic curve (AUC) for spiked vs non-spiked p values from differential relative abundance (DA) tests. AUC distributions from 150 iterations for each case proportion (vertical panels) in dataset A4, analyzed with all differential relative abundance methods (horizontal panels). Black dots represent medians in each distribution. (ZIP 1831 kb) [file 40168_2016_208_MOESM8_ESM.zip › S7A_Figure.pdf]

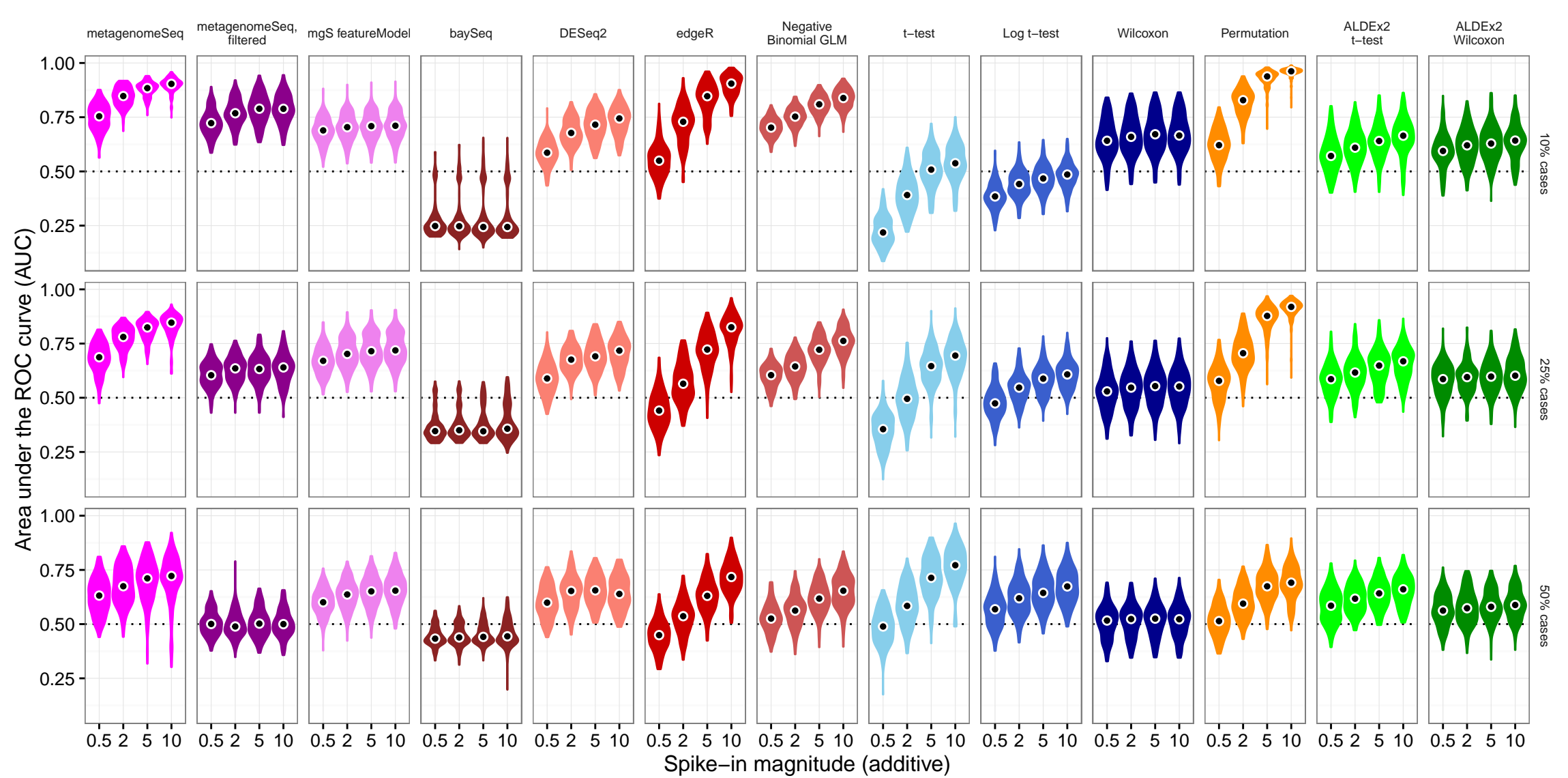

Supplement: Additional file 8: Figure S7. — A. Area under the curve distributions for multiplicative spike-ins in dataset A4. Violin plot of distributions of area under the receiver operating characteristic curve (AUC) for spiked vs non-spiked p values from differential relative abundance (DA) tests. AUC distributions from 150 iterations for each combination of multiplicative spike-in magnitude and case proportion (vertical panels) in dataset A4, analyzed with all differential relative abundance methods (horizontal panels). Black dots represent medians in each distribution. B. Area under the curve distributions for additive spike-ins in dataset A4. Violin plot of distributions of area under the receiver operating characteristic curve (AUC) for spiked vs non-spiked p values from differential relative abundance (DA) tests. AUC distributions from 150 iterations for each combination of additive spike-in magnitude and case proportion (vertical panels) in dataset A4, analyzed with all differential relative abundance methods (horizontal panels). Black dots represent medians in each distribution. C. Area under the curve distributions for mixed multiplicative spike-ins in dataset A4. Violin plot of distributions of area under the receiver operating characteristic curve (AUC) for spiked vs non-spiked p values from differential relative abundance (DA) tests. AUC distributions from 150 iterations for each case proportion (vertical panels) in dataset A4, analyzed with all differential relative abundance methods (horizontal panels). Black dots represent medians in each distribution. (ZIP 1831 kb) [file 40168_2016_208_MOESM8_ESM.zip › S7B_Figure.pdf]

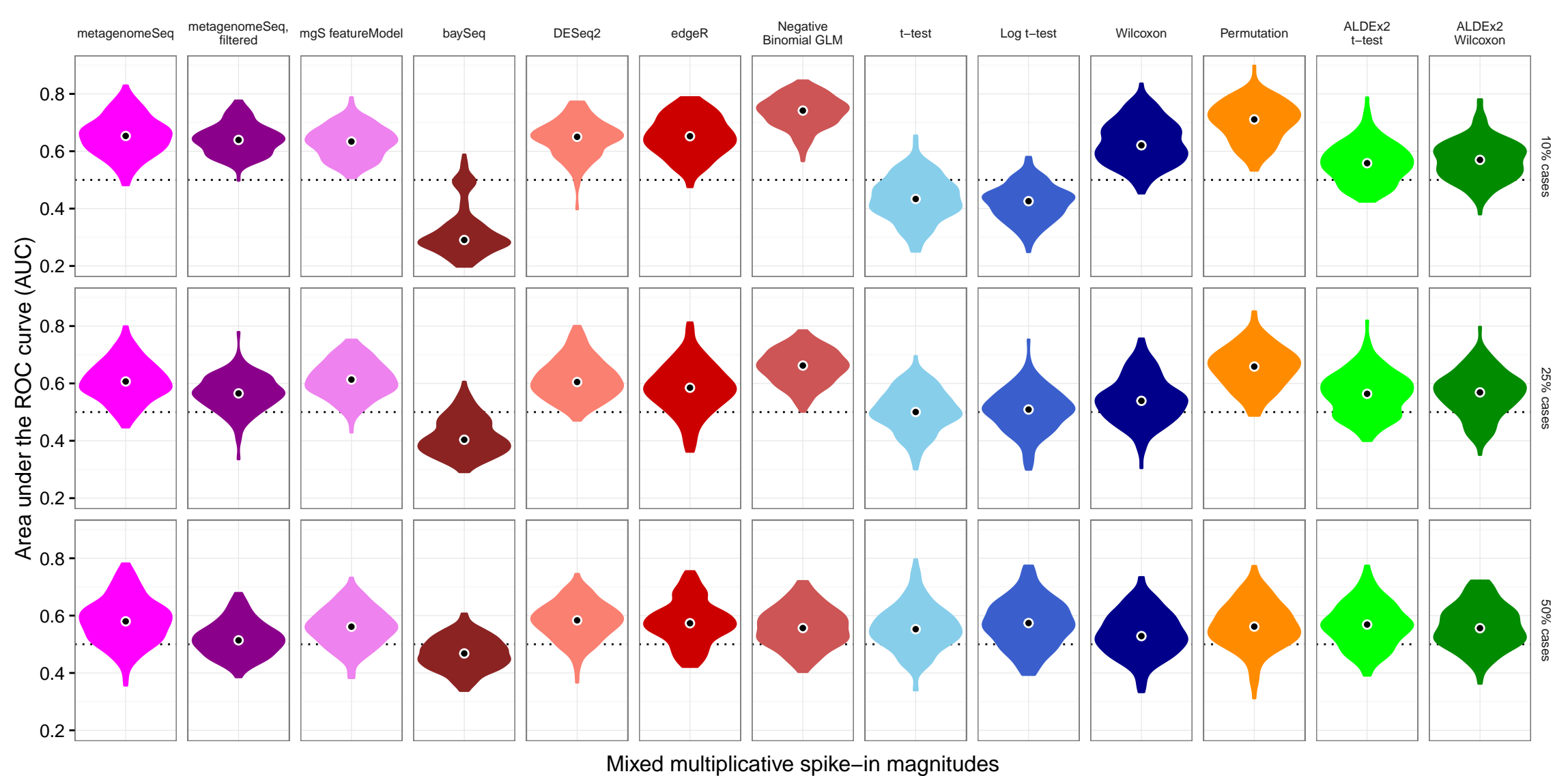

Supplement: Additional file 8: Figure S7. — A. Area under the curve distributions for multiplicative spike-ins in dataset A4. Violin plot of distributions of area under the receiver operating characteristic curve (AUC) for spiked vs non-spiked p values from differential relative abundance (DA) tests. AUC distributions from 150 iterations for each combination of multiplicative spike-in magnitude and case proportion (vertical panels) in dataset A4, analyzed with all differential relative abundance methods (horizontal panels). Black dots represent medians in each distribution. B. Area under the curve distributions for additive spike-ins in dataset A4. Violin plot of distributions of area under the receiver operating characteristic curve (AUC) for spiked vs non-spiked p values from differential relative abundance (DA) tests. AUC distributions from 150 iterations for each combination of additive spike-in magnitude and case proportion (vertical panels) in dataset A4, analyzed with all differential relative abundance methods (horizontal panels). Black dots represent medians in each distribution. C. Area under the curve distributions for mixed multiplicative spike-ins in dataset A4. Violin plot of distributions of area under the receiver operating characteristic curve (AUC) for spiked vs non-spiked p values from differential relative abundance (DA) tests. AUC distributions from 150 iterations for each case proportion (vertical panels) in dataset A4, analyzed with all differential relative abundance methods (horizontal panels). Black dots represent medians in each distribution. (ZIP 1831 kb) [file 40168_2016_208_MOESM8_ESM.zip › S7C_Figure.pdf]

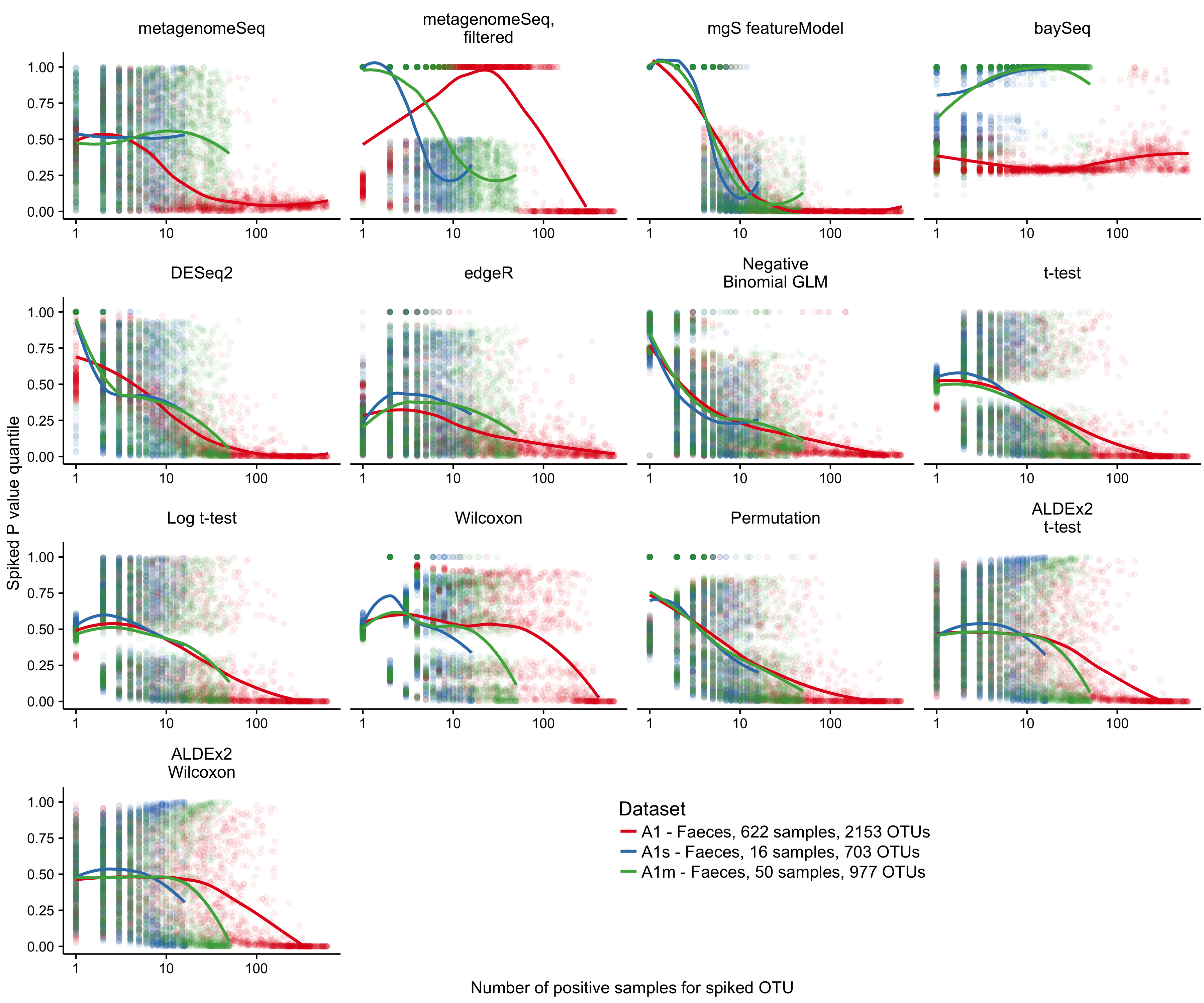

Supplement: Additional file 9: Figure S8. — A. Spike-in retrieval as a function of number of positive samples, by dataset size. Aggregated results across 150 iterations of multiplicative spike-ins of magnitude 5 with 50% cases, in datasets A1, A1s, and A1m. Each dot represents a spiked OTU. The Y-axis displays its p value quantile (0 is lowest p value, 1 is highest p value) within that DA run, and the X axis shows how many samples are positive (nonzero) for that OTU. Results from the three datasets are overlaid with different colors and faceted by statistical method. B. Spike-in retrieval as a function of number of positive samples, by case proportion. Aggregated results across 150 iterations of multiplicative spike-ins of magnitude 5 with 10, 25, or 50% cases, in dataset A1. Each dot represents a spiked OTU. The Y-axis displays its p value quantile (0 is lowest p value, 1 is highest p value) within that DA run, and the X axis shows how many samples are positive (nonzero) for that OTU. Results from the three case proportions are overlaid with different colors, and faceted by statistical method. C. Spike-in retrieval as a function of number of positive samples, by spike-in magnitude. Aggregated results across 150 iterations of multiplicative spike-ins of magnitudes 0.5, 2, 5, 10, and 20 with 50% cases, in dataset A1. Each dot represents a spiked OTU. The Y-axis displays its p value quantile (0 is lowest p value, 1 is highest p value) within that DA run, and the X axis shows how many samples are positive (nonzero) for that OTU. Results from the different spike-in magnitudes are overlaid with different colors, and faceted by statistical method. (ZIP 16398 kb) [file 40168_2016_208_MOESM9_ESM.zip › S8A_Figure.png]

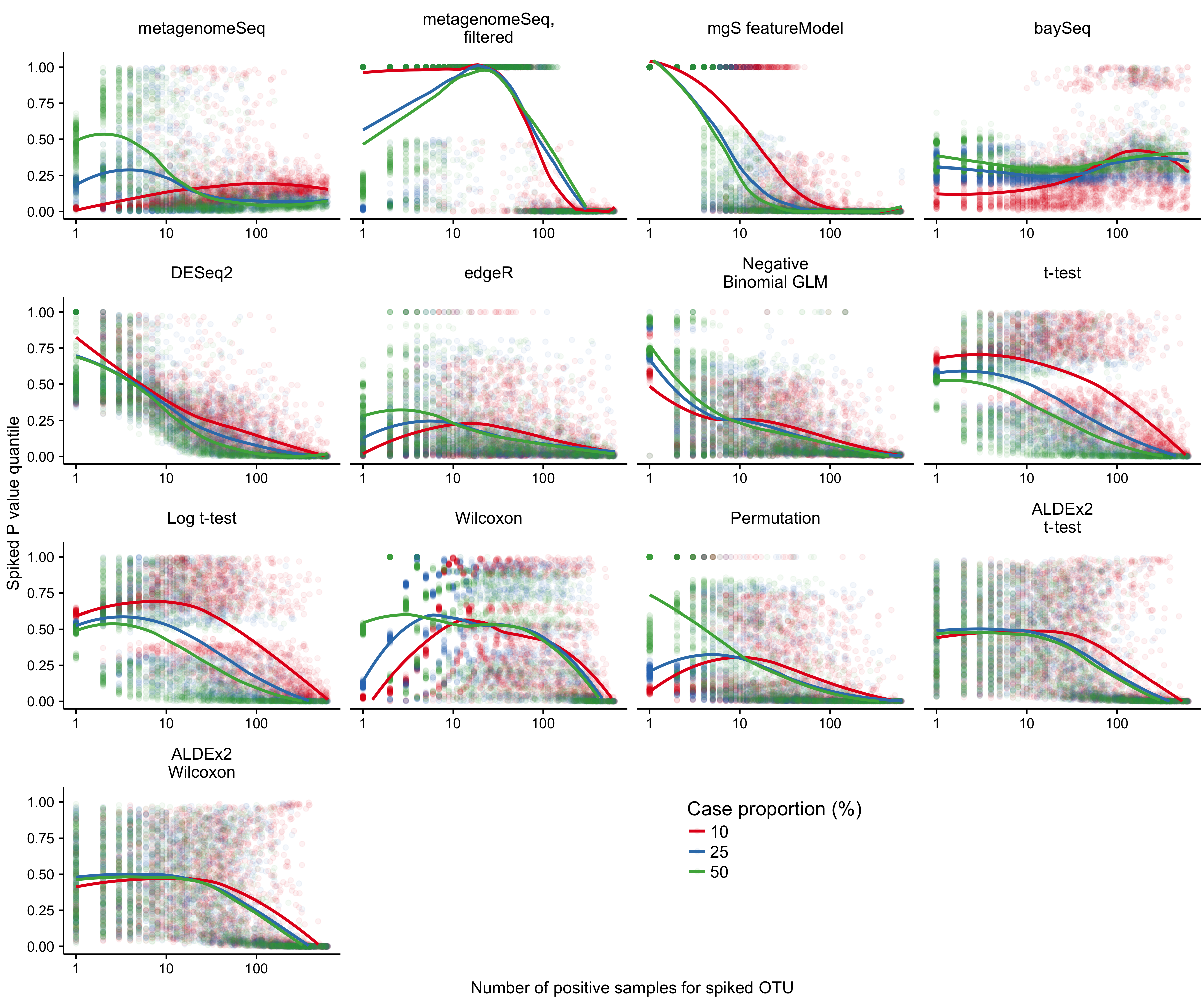

Supplement: Additional file 9: Figure S8. — A. Spike-in retrieval as a function of number of positive samples, by dataset size. Aggregated results across 150 iterations of multiplicative spike-ins of magnitude 5 with 50% cases, in datasets A1, A1s, and A1m. Each dot represents a spiked OTU. The Y-axis displays its p value quantile (0 is lowest p value, 1 is highest p value) within that DA run, and the X axis shows how many samples are positive (nonzero) for that OTU. Results from the three datasets are overlaid with different colors and faceted by statistical method. B. Spike-in retrieval as a function of number of positive samples, by case proportion. Aggregated results across 150 iterations of multiplicative spike-ins of magnitude 5 with 10, 25, or 50% cases, in dataset A1. Each dot represents a spiked OTU. The Y-axis displays its p value quantile (0 is lowest p value, 1 is highest p value) within that DA run, and the X axis shows how many samples are positive (nonzero) for that OTU. Results from the three case proportions are overlaid with different colors, and faceted by statistical method. C. Spike-in retrieval as a function of number of positive samples, by spike-in magnitude. Aggregated results across 150 iterations of multiplicative spike-ins of magnitudes 0.5, 2, 5, 10, and 20 with 50% cases, in dataset A1. Each dot represents a spiked OTU. The Y-axis displays its p value quantile (0 is lowest p value, 1 is highest p value) within that DA run, and the X axis shows how many samples are positive (nonzero) for that OTU. Results from the different spike-in magnitudes are overlaid with different colors, and faceted by statistical method. (ZIP 16398 kb) [file 40168_2016_208_MOESM9_ESM.zip › S8B_Figure.png]

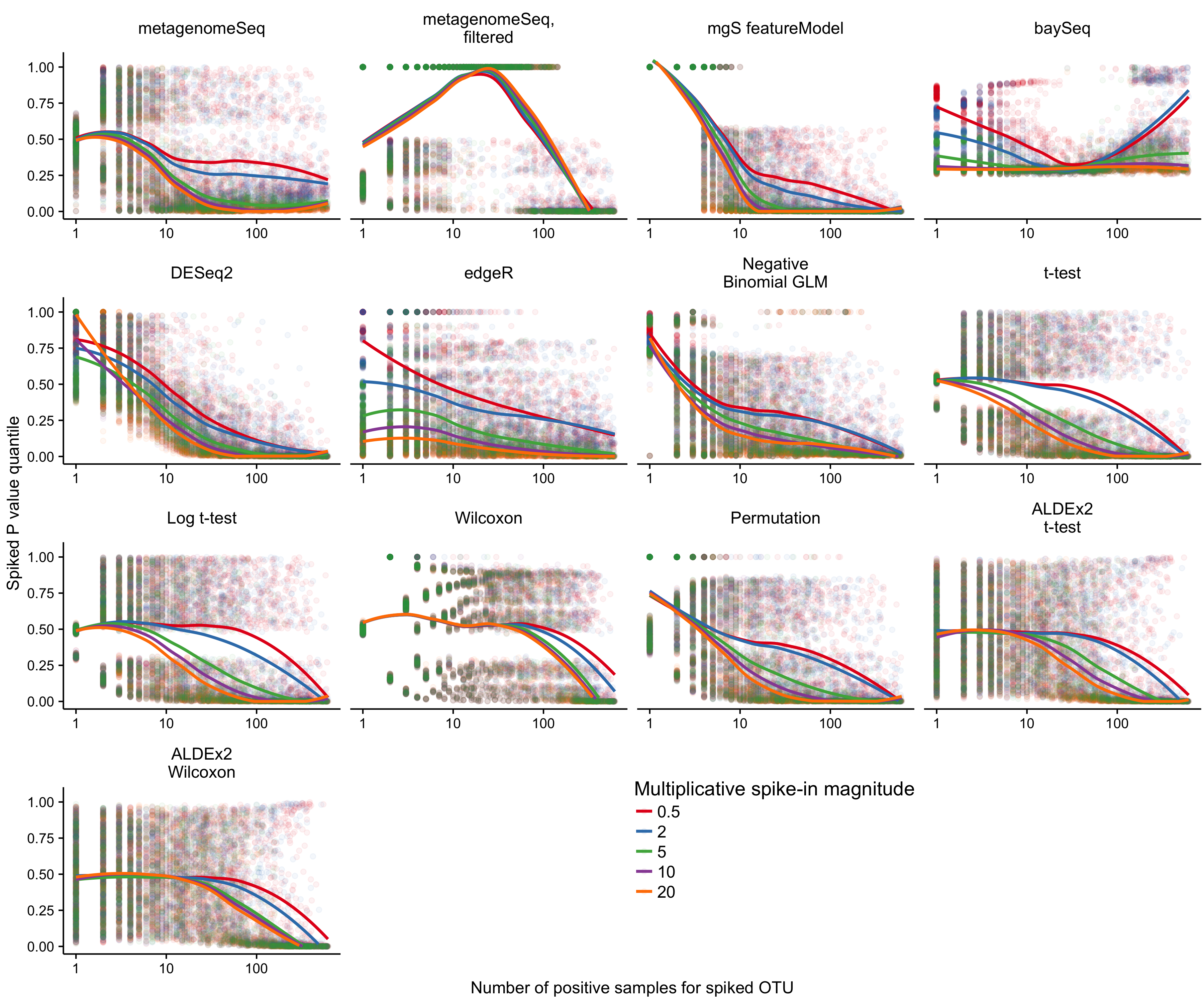

Supplement: Additional file 9: Figure S8. — A. Spike-in retrieval as a function of number of positive samples, by dataset size. Aggregated results across 150 iterations of multiplicative spike-ins of magnitude 5 with 50% cases, in datasets A1, A1s, and A1m. Each dot represents a spiked OTU. The Y-axis displays its p value quantile (0 is lowest p value, 1 is highest p value) within that DA run, and the X axis shows how many samples are positive (nonzero) for that OTU. Results from the three datasets are overlaid with different colors and faceted by statistical method. B. Spike-in retrieval as a function of number of positive samples, by case proportion. Aggregated results across 150 iterations of multiplicative spike-ins of magnitude 5 with 10, 25, or 50% cases, in dataset A1. Each dot represents a spiked OTU. The Y-axis displays its p value quantile (0 is lowest p value, 1 is highest p value) within that DA run, and the X axis shows how many samples are positive (nonzero) for that OTU. Results from the three case proportions are overlaid with different colors, and faceted by statistical method. C. Spike-in retrieval as a function of number of positive samples, by spike-in magnitude. Aggregated results across 150 iterations of multiplicative spike-ins of magnitudes 0.5, 2, 5, 10, and 20 with 50% cases, in dataset A1. Each dot represents a spiked OTU. The Y-axis displays its p value quantile (0 is lowest p value, 1 is highest p value) within that DA run, and the X axis shows how many samples are positive (nonzero) for that OTU. Results from the different spike-in magnitudes are overlaid with different colors, and faceted by statistical method. (ZIP 16398 kb) [file 40168_2016_208_MOESM9_ESM.zip › S8C_figure.png]

**A**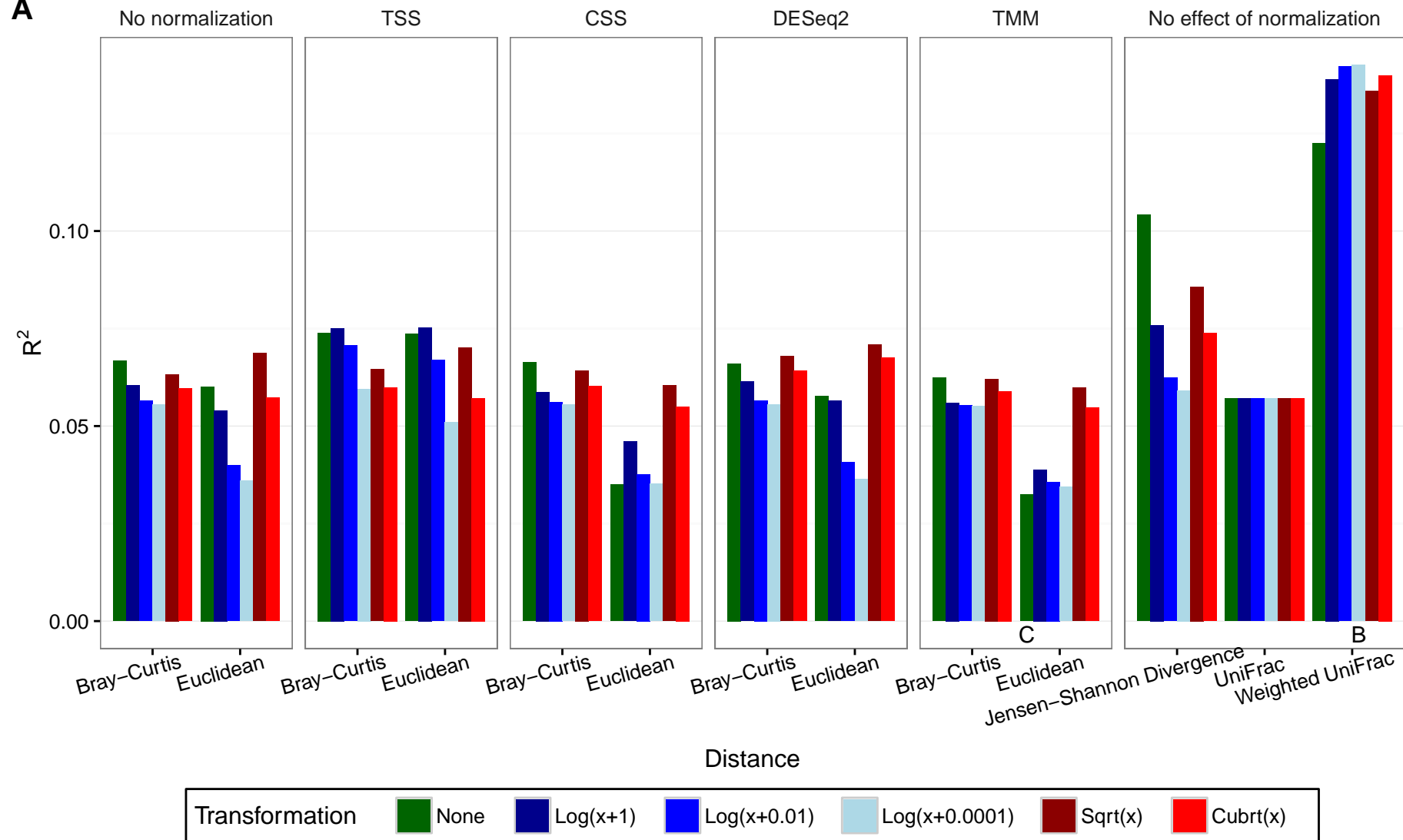**B**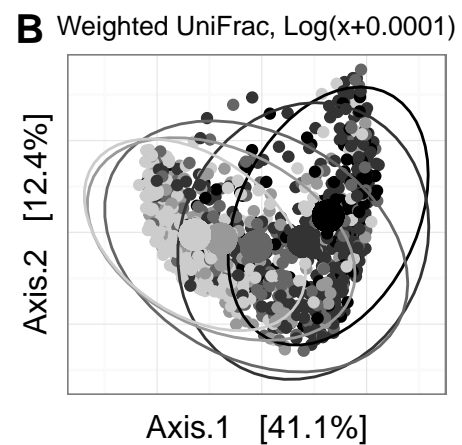**C**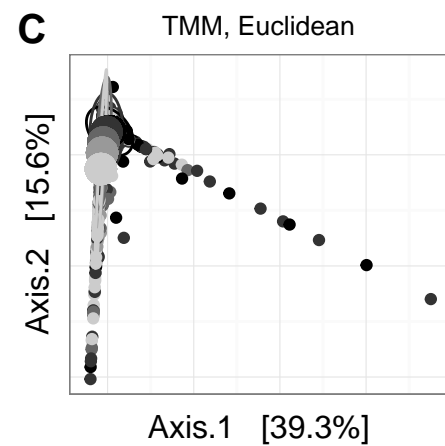

Supplement: Additional file 10: Figure S9. — The effect of normalization, transformation, and distance metric on beta-diversity separation in the modified dataset B3. Dataset B3 modified by grouping of several OTUs, reducing the dataset to 8770 OTUs. All combinations of library size normalizations (horizontal panels) and count transformations (color) applied prior to calculation of distances and use of Adonis permanova model. Effect of age group measured as model R 2 value. The highest and lowest R 2 values (yielding best and worst separation, respectively) are demonstrated in subplots B & C as principal coordinate analysis plots, colored by design variable, with overlaid prediction ellipses. CSS cumulative sum scaling, TMM trimmed mean of M values, TSS total sum scaling. (PDF 124 kb) [file 40168_2016_208_MOESM10_ESM.pdf]

**Most differentiated wilcoxon statistics (under H0)**

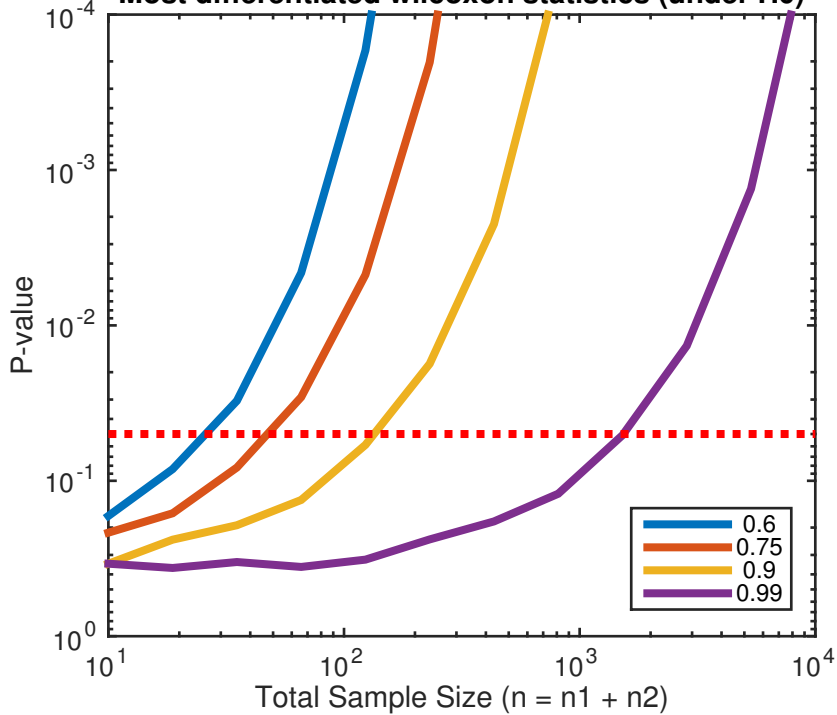

Supplement: Additional file 12: Figure S10. — The lowest obtainable p values from a Wilcoxon rank sum test depend on sample size and sparsity. Overview of the lowest p value theoretically obtainable using optimal conditions for a given sample size and level of sparsity. Optimal conditions refer to (a) 50% cases, i.e., n1 = n2, (b) only nonzero counts in one of the groups, limited by level of sparsity, (c) no ties between nonzero counts. One sided p values from normal approximation shown. Since the p value only depends on the rank distribution, the actual count values do not matter. Color of the lines indicates level of sparsity; dotted red line corresponds to p = 0.05. (PDF 9 kb) [file 40168_2016_208_MOESM12_ESM.pdf]
