# Supplementary material for: Large-scale benchmarking reveals false discoveries and count transformation sensitivity in 16S rRNA gene amplicon data analysis methods used in microbiome studies
Source: Microbiome. 2016 Nov 25;4:62. doi: 10.1186/s40168-016-0208-8 (PMC5123278; doi:10.1186/s40168-016-0208-8)
Supplement: Additional file 11: Table S3. — Summary of benchmark performance for included tools. (PDF 32 kb) [file 40168_2016_208_MOESM11_ESM.pdf]

T-test	- Assumes a gaussian distribution
	- FPR: Robust across the range
	- AUC performance: Low
	- AUC robustness: Low
	- Balance sensitivity: Sensitive to balanced studies
	- Computational burden: very fast
Log t-test	- Assumes a log-gaussian distribution
	- FPR: Robust across the range
	- AUC performance: Low
	- AUC robustness: Low
	- Balance sensitivity: Sensitive to balanced studies
	- Computational burden: very fast
Wilcoxon rank sum test	- No distributional assumptions, uses ranks
	- FPR: Robust across the range
	- AUC performance: Low
	- AUC robustness: Low
	- Balance sensitivity: Low
	- Computational burden: Very fast
Negative binomial GLM	- Assumes a negative binomial distribution
	- FPR: Robust, conservative with high sparsity
	- AUC performance: Medium
	- AUC robustness: High
	- Balance sensitivity: Low
	- Computational burden: Medium
DEseq2	- Assumes a negative binomial distribution

	- FPR: Robust, very conservative with high sparsity
	- AUC performance: Medium
	- AUC robustness: Medium
	- Balance sensitivity: Low
	- Computational burden: Slow
edgeR	- Assumes a negative binomial distribution
	- FPR: Inflated
	- AUC performance: Good
	- AUC robustness: Medium
	- Balance sensitivity: Low
	- Computational burden: Medium
baySeq	- Assumes a negative binomial distribution, bayesian inference
	- FPR: Inflated
	- AUC performance: Good
	- AUC robustness: Medium
	- Balance sensitivity: Low
	- Computational burden: Very slow
ALDEx2	- Assumes gaussian (t-test) or no assumptions (Wilcoxon) when testing Dirichlet Monte Carlo samples
	- FPR: Conservative
	- AUC performance: Medium
	- AUC robustness: Medium
	- Balance sensitivity: Low
	- Computational burden: Very slow
metagenomeSeq ZIG, filtered	- Assumes a log-gaussian distribution
	- FPR: Slightly inflated

	- AUC performance: Good
	- AUC robustness: Medium
	- Balance sensitivity: Sensitive to unbalanced studies
	- Computational burden: Fast
metagenomeSeq, featureModel	- Assumes a log-gaussian distribution
	- FPR: Robust, very conservative with high sparsity
	- AUC performance: Good
	- AUC robustness: High
	- Balance sensitivity: Low
	- Computational burden: Fast
Permutation test	- No distributional assumptions
	- FPR: Robust across the range
	- AUC performance: Medium
	- AUC robustness: High
	- Balance sensitivity: Low
	- Computational burden: Slow, depending on number of permutations
