# Supplementary material for: Large-scale benchmarking reveals false discoveries and count transformation sensitivity in 16S rRNA gene amplicon data analysis methods used in microbiome studies
Source: Microbiome. 2016 Nov 25;4:62. doi: 10.1186/s40168-016-0208-8 (PMC5123278; doi:10.1186/s40168-016-0208-8)
Supplement: Additional file 13: — Supplementary methods. (DOCX 15 kb) [file 40168_2016_208_MOESM13_ESM.docx]

Additional file 13

## DNA extraction [Dataset A1, A2, A3, B1]

Genomic DNA was extracted from the samples using the PowerMag® Soil DNA Isolation Kit Optimized for epMotion® (MO-BIO Laboratories, Inc., Carlsberg, CA, US), using the epMotion® robotic platform model (Eppendorf). The following alterations were made to the the manufacturer’s protocol: 150 µl of the samples were added to the 96-well bead plate, containing 750 µl bead/RNase A Solution and 60 µl lysis solution. Centrifugation steps were performed at 3220xRCF for 9 minutes. Removal of enzymatic inhibitors and DNA purification was performed as described by the manufacturer. Finally, the DNA was eluted with 100 µl Tris buffer (10mM, pH 7.5). DNA concentrations were determined using the Quant-iTTM PicoGreen® quantification system (Life Technologies, CA, US). Extracted DNA was stored at -20°C.

## 16S amplicon sequencing [Dataset A1, A2, A3, B1]

The 16S rDNA amplification procedure was divided into two PCR steps. In the first PCR reaction, the hypervariable V4 region of the 16S rRNA gene was amplified using the broad range primers 515F (5’-GTGCCAGCMGCCGCGGTAA-3’) and 806R modified from Sundberg et al. (5’-GGACTACHVGGGTWTCTAAT-3’)[1–3]. Amplification was performed in 96-well microtiter plates with a reaction mixture consisting of 1X AccuPrime PCR Buffer II, 0.6 U AccuPrime Taq DNA Polymerase (Invitrogen, Life technologies, CA, US), 0.5 µM primer 515F, 0.5 µM primer 806R, and 2.0 µl template DNA, giving a total volume of 20.0 µl per sample. Reactions were run in a 2720 thermal cycler (Applied Biosystems®, Life Technologies, CA, US) according to the following cycling program: 2 minutes of denaturation at 94°C, followed by 30 cycles of 20 seconds at 94°C (denaturing), 30 seconds at 56°C (annealing) and 40 seconds at 68°C (elongation), with a final extension at 68°C for 5 minutes. For each plate, a negative template-free control and a positive control containing 2.0 µl DNA from a known bacterial mock community (1.0 ng/µl; HM-782D, BEI Resources, VA, US) were included. The PCR products were quantified using the Quant-iT PicoGreen quantification system (Life Technologies, CA, US) and samples with a concentration above 6.0 ng/µl were diluted to approximately 3.0-6.0 ng/µl prior to further analysis.

Sequencing primers and adaptors were added to the amplicon products in the second PCR step as follows: 2.0 µl of the diluted amplicons were mixed with a reaction solution consisting of 1X AccuPrime PCR Buffer II, 0.6U AccuPrime Taq DNA Polymerase (Invitrogen, Life technologies, CA, US) and 0.5 µM fusion forward (10.0 µM) and 0.5 µM fusion reverse primer (total volume: 20 µl). The PCR was run according to the cycling program above, except with a reduced cycling number of 15. The amplification products were purified with Agencourt AMPure XP Beads (Beckman Coulter Genomics, MA, US) according to the manufacturer’s specifications and using 0.7X volume beads, and was quantified as described above. Equimolar amounts of the amplification products were pooled together in a single tube. The pooled DNA samples were concentrated using the DNA Clean & Concentrator TM-5 Kit (Zymo Research, Irvine, CA, US) according to the manufacturer’s instructions. The concentration of the pooled libraries was determined using the Quant-iT™ High-Sensitivity DNA Assay Kit (Life Technologies) following the specifications of the manufacturer. Amplicon sequencing was performed on the Illumina MiSeq Desktop Sequencer (Illumina Inc., CA, US). For each run, a 1.0%-5% PhiX internal control was included. All reagents used were from the MiSeq Reagent Kit v2 (Illumina Inc., CA, US). Automated cluster generation and 250 paired-end sequencing with dual-index reads were performed. The sequencing output was generated as demultiplexed FASTQ-files for downstream analysis. Up to 192 samples were sequenced per run.

## Bioinformatics [Dataset A1, A2, A3, B1]

FASTQ files demultiplexed by the MiSeq Controller Software was trimmed for diversity spacers using biopieces [4]. Read pairs were mated using USEARCH v7.0.1090 [5] and quality-filtered using USEARCH -maxee 0.5 to remove reads with expected errors higher than 0.5. UPARSE [6] was used for OTU clustering as recommended, in particular removing singletons after dereplication. Chimera checking was performed with USEARCH against the gold database [7]. Representative sequences of each OTU were classified using Mothur v.1.25.0 wang() function [8] at 0.8 confidence threshold. Qiime wrappers for PyNAST [9], FastTree[10], and filter_alignment.py [11] were used to construct a phylogenetic tree. Alignments were built against the 2011 version of Greengenes [12] and filtered using --allowed_gap_frac 0.999999 and --threshold 3.0, the QIIME defaults

## Supplemental References

1. Neefs JM, De Wachter R. A proposal for the secondary structure of a variable area of eukaryotic small ribosomal subunit RNA involving the existence of a pseudoknot. Nucleic Acids Res. 1990;18: 5695–5704.

2. Yu Y, Lee C, Kim J, Hwang S. Group-specific primer and probe sets to detect methanogenic communities using quantitative real-time polymerase chain reaction. Biotechnol Bioeng. 2005;89: 670–679. doi:10.1002/bit.20347

3. Sundberg C, Al-Soud WA, Larsson M, Alm E, Yekta SS, Svensson BH, et al. 454 pyrosequencing analyses of bacterial and archaeal richness in 21 full-scale biogas digesters. FEMS Microbiol Ecol. 2013;85: 612–626. doi:10.1111/1574-6941.12148

4. Biopieces.org [Internet]. [cited 5 Oct 2015]. Available: https://github.com/maasha/biopieces

5. Edgar RC. Search and clustering orders of magnitude faster than BLAST. Bioinforma Oxf Engl. 2010;26: 2460–2461. doi:10.1093/bioinformatics/btq461

6. Edgar RC. UPARSE: highly accurate OTU sequences from microbial amplicon reads. Nat Methods. 2013;10: 996–998. doi:10.1038/nmeth.2604

7. Haas BJ, Gevers D, Earl AM, Feldgarden M, Ward DV, Giannoukos G, et al. Chimeric 16S rRNA sequence formation and detection in Sanger and 454-pyrosequenced PCR amplicons. Genome Res. 2011;21: 494–504. doi:10.1101/gr.112730.110

8. Schloss PD, Westcott SL, Ryabin T, Hall JR, Hartmann M, Hollister EB, et al. Introducing mothur: open-source, platform-independent, community-supported software for describing and comparing microbial communities. Appl Environ Microbiol. 2009;75: 7537–7541. doi:10.1128/AEM.01541-09

9. Caporaso JG, Bittinger K, Bushman FD, DeSantis TZ, Andersen GL, Knight R. PyNAST: a flexible tool for aligning sequences to a template alignment. Bioinforma Oxf Engl. 2010;26: 266–267. doi:10.1093/bioinformatics/btp636

10. Price MN, Dehal PS, Arkin AP. FastTree: computing large minimum evolution trees with profiles instead of a distance matrix. Mol Biol Evol. 2009;26: 1641–1650. doi:10.1093/molbev/msp077

11. Caporaso JG, Kuczynski J, Stombaugh J, Bittinger K, Bushman FD, Costello EK, et al. QIIME allows analysis of high-throughput community sequencing data. Nat Methods. 2010;7: 335–336. doi:10.1038/nmeth.f.303

12. greengenes.lbl.gov - Aligned 16S rDNA data and tools [Internet]. [cited 5 Oct 2015]. Available: http://greengenes.lbl.gov/
